# Optimizing cognitive load in Guzheng learning: a narrative review of demonstration, segmented practice, and feedback scaffolding

**DOI:** 10.3389/fpsyg.2026.1881131

**Published:** 2026-07-09

**Authors:** Yumeng Wu

**Affiliations:** Heilongjiang University, Harbin, China

**Keywords:** cognitive load theory, demonstration, educational psychology, feedback scaffolding, Guzheng learning, instructional design, segmented practice, working memory

## Abstract

Learning to play the Guzheng requires learners to coordinate notation, motor control, auditory monitoring, and musical interpretation under substantial attentional constraints. Framed by Cognitive Load Theory (CLT), this narrative review examines how cognitive load may be better managed in Guzheng learning, with particular attention to demonstration, segmented practice, and feedback scaffolding. Literature was identified through Web of Science Core Collection and Scopus, with a main search window from 1 January 2019 to 22 December 2025; earlier studies were retained selectively when foundational to CLT or specific instructional mechanisms. Greater interpretive weight was given to direct Guzheng evidence where available, followed by broader music and instrumental-learning research, while adjacent complex-skill studies were used more cautiously for mechanism-level interpretation. Across the reviewed literature, cognitive load appears to be shaped by both intrinsic task complexity and avoidable extraneous demands, especially split attention, poorly coordinated multimedia presentation, attentional switching during demonstrations, and redundant digital input. Integrated notation, segmented rehearsal, worked examples with fading support, and calibrated feedback appear pedagogically promising, but direct Guzheng-specific evidence remains limited. More research is needed on retention, transfer, learner variation, and the validation of instructional designs grounded in cognitive and educational psychology within Guzheng education.

## 1 Introduction

The study of learning how to play the Guzheng, an ancient Chinese plucked string instrument with over 2,500 years of tradition, presents an interesting instance of multifaceted skill acquisition. Learners need to be able to decipher notation, keep track of rhythm and pitch, control fine motor movement, and create musical expression in real time in order to perform successfully. These demands are not sequential in the case of beginners, but rather they are competing for restricted attentional resources that frequently lead to overload, which interferes with the first steps as well as subsequent memorization. So, the process of learning Guzheng is not merely a question of musical practice but also of instructional design.

Cognitive Load Theory (CLT) can be used as an effective model to solve such a problem. Being a long-standing viewpoint in the field of educational psychology, CLT describes the relationship between task variables and instructional design and the capacity of working memory. Learners may spend much time processing irrelevant information or badly organized information instead of developing knowledge structures that can last longer when the instructional materials are badly sequenced, too dense, or hard to integrate. Instruction that controls the level of complexity of tasks and minimizes unnecessary processing requirements is also more likely to facilitate stable schema formation and transfer. They are particularly acute in Guzheng education, where notation, fingerings, auditory monitoring, and bimanual coordination are frequently needed at the same time.

CLT is also relevant when Guzheng instruction extends beyond conventional face-to-face teaching. Video demonstrations, interactive notation, platform-based practice, and AI- or VR-supported tools may increase access and practice flexibility. However, they may also add extraneous load when visual, auditory, and textual information is poorly coordinated. Their instructional value, therefore, depends on how they organize attention and support learning rather than on technological novelty alone.

Against this background, the present narrative review examines cognitive load optimization in Guzheng learning through three instructional domains: demonstration, segmented practice, and feedback scaffolding. It synthesizes recent evidence from Guzheng-related studies, broader music and instrumental-learning research, and selected adjacent complex-skill fields, while retaining foundational CLT sources where necessary. The review aims to identify plausible instructional mechanisms, clarify the limits of the current evidence, and outline priorities for future research and instructional design.

### 1.1 Cognitive load theory as a framework for instructional design

Cognitive Load Theory (CLT) was developed in the 1980s as an instructional design theory based on prior descriptions of human cognitive structure (Sweller et al., 2019). One of the fundamental assumptions of CLT is that learning is determined by the interplay of two memory systems, that is, a working memory, which has a limited capacity to process new information, and a long-term memory that can retain knowledge in a form that would be helpful in the future to retrieve it effectively and use it efficiently (Sweller et al., 2019). Sweller et al. (1998) also added that the extreme limitations of working memory can be overcome when knowledge is structured into schemas in long-term memory, thus allowing learners to execute tasks they could not perform in their original state (Sweller et al., 2019). With time, CLT has turned out to be one of the most popular frameworks to justify why certain kinds of instructions make learning easier, whereas others make it more difficult (Sweller et al., 2019).

In CLT, cognitive load is usually classified as three related types: intrinsic, extraneous, and germane load (Sweller et al., 2019). The intrinsic cognitive load comes about as a result of the natural complexity of the material, particularly when several interdependent components need to be considered simultaneously. With regard to Guzheng education, this might involve synchronizing the actions of both hands as well as following finger patterns, changes in pitch, rhythmic patterns, and expressiveness. Conversely, extraneous cognitive load is caused by aspects of instructional design that are not directly helpful to learning. Practically, it happens when notation appears to be divided visually, when students have to constantly switch their attention between score symbols and performance markings, or when redundant explanations are competing with limited working-memory space. The term Germane cognitive load is used to represent an effort aimed at schema construction and automation- the type of mental labor that has a meaningful contribution to learning (Sweller et al., 2019). According to the CLT view, successful teaching is not merely a reduction in overall mental effort but a control of intrinsic complexity, the elimination of unnecessary extraneous requirements, and the encouragement of the type of processing which enables students to develop transferable skills (Sweller et al., 2019).

The concept is particularly useful in technology-intensive teaching environments. The increasing number of digital devices employed in the educational environment has transformed the way information is delivered, practiced, and revisited, which is why the creation of learning spaces becomes more important (Skulmowski and Xu, 2022). Accessibility and engagement in digital Guzheng instruction may be enhanced by the use of multimedia resources, including annotated video demonstrations, interactive notation, and virtual feedback interfaces. They can also add extra processing requirements if visual, auditory, and textual cues are not well coordinated (Skulmowski and Xu, 2022). A mixed methods study of CLT in digital classrooms found that less integrated multimedia materials were related to greater cognitive load, but segmentation and simplification of the materials helped in improving learning results (Skulmowski and Xu, 2022). The results are directly applicable to the field of Guzheng pedagogy, as the learning process often relies on coordinating different data flows instead of processing them separately.

Even though most of the CLT literature is written by authors who are not connected to the field of music education, its fundamental ideas can be helpful in Guzheng learning since they focus on the process through which novices learn demanding procedural skills. To illustrate this point in the context of medical education, a quasi-experimental study established that an instruction design based on CLT reduced overall cognitive load and enhanced the performance of students in learning clinical procedures (Wasfy et al., 2021). Surgical training involved the use of expert feedback at the beginning of practice that minimized cognitive load and facilitated combining declarative knowledge and physical execution (Cecilio-Fernandes et al., 2020). These similarities are not identical, but they remain insightful. Learning Guzheng also involves the coordination of bodily movement, perceptual discrimination, and rule-based performance with limited cognitive capacity.

CLT can also be enhanced with viewpoints that can deal with how technology and embodiment can influence learning. An example of this is the Cognitive Affective Model of Immersive Learning (CAMIL), which integrates literature on immersive virtual reality in demonstrating the way affordances like presence and agency relate to cognitive load and affect learning outcomes (Makransky and Petersen, 2021). Immersive technologies have potential in Guzheng education to provide valuable opportunities to undertake guided practice, but it requires careful calibration; it is possible that novelty or interface complexity becomes a source of extraneous load (Makransky and Petersen, 2021). Similarly, embodied and enactive descriptions of cognition highlight the idea that learning is not limited to mental abstraction processes alone. Practical interaction with an instrument, such as tactile response to strings and the repeated movements of a physical pattern, may shift certain requirements that would have otherwise been internalized and require effort (Gallagher, 2023). All these views support the necessity to consider Guzheng learning not merely as a musical activity but also as a cognitively and materially contextualized activity.

### 1.2 Guzheng learning as a complex skill context

The guzheng (21 or more stringed), with a range exceeding three octaves, provides students with a unique challenge in musical, perceptual, and motor terms. In contrast to easier instrumental skills, playing the Guzheng usually involves a large amount of bimanual coordination. The right hand typically plays the melodic line by plucking patterns, whereas the left hand changes the pitch, timbre, and expressive contour by pressure vibrato and similar methods. All these actions should be time-synchronized. To the novice learner, this involves the control of independent yet interrelated information streams as well as the monitoring of sound quality and interpretation of scores. These circumstances put constant strain on working memory and make the study of Guzheng especially important as an example of CLT-focused research (Cole and Shields, 2019).

Its challenge is also in its requirement to relate various kinds of knowledge. Children should integrate declarative information (notation systems, string labels, and fundamental musical notions) and procedural information (hand placement, plucking patterns, and emotional regulation). The perceptual-motor skills, including the ability to discriminate the pitch and the accuracy of rhythm and monitor sensory feedback, evolve with those knowledge systems, not after them. Studies on the development of motor skills have demonstrated that higher cognitive loads will slow learning, decrease the application of learning to novel contexts, and raise errors during unpredictable or unexpected task requirements (Cole and Shields, 2019). One study using a weight-bearing cognitive-motor dual-task found that executive function explained a significant portion of performance variance, which suggests that the coordination of multiple demands requires the involvement of higher-order cognitive control mechanisms (Cole and Shields, 2019). The message to Guzheng learners is simple: if attention control or working memory is problematic, it is going to affect how well they learn right away.

Nevertheless, musical training is not just demanding on a cognitive level; it can be developmental as well. A randomized controlled study with primary school children demonstrated that class-based formal string instrument training was associated with enhanced working memory, attention, processing speed, cognitive flexibility, and bimanual coordination in comparison to traditional music sensitization programs (James et al., 2020). Similarly, studies of older adults who learned to play the piano demonstrated the improvement of fine motor skills, working memory, and processing speed, as well as structural brain plasticity in areas related to motor processes (Worschech et al., 2023). These results are part of more general findings regarding music learning, but they reinforce the idea that focused instrumental practice has significant cognitive and motor gains with time. The possible benefits of such education in Guzheng education are contingent on how early learning is organized so that complexity becomes manageable instead of overwhelming.

The educational setting is also important. The cognitive load is influenced not just by the tool and the activity, but by the social and physical situations in which practice takes place. In typical classrooms, irrelevant interruptions, unproductive practice schedules, or emotionally laden feedback can add to extraneous load since it shifts focus off task-related thoughts (Babchenko et al., 2019). Studies on surgical training have demonstrated that negative learning conditions have an effect on performance since the emotion interferes with the working memory sources (Babchenko et al., 2019). An equivalent process could be likely in the case of Guzheng instruction. Students who are required to manage anxiety, embarrassment, or over-monitoring their performance could have less capacity to control and interpret music technically. By contrast, an encouraging setting that regards challenge as a healthy aspect of learning will tend to help maintain attentional space and motivation (Babchenko et al., 2019).

Such concerns are even more relevant when speaking about contexts mediated by digital technologies. Instruction with Guzheng could be more flexible and available through video instruction, practice on platforms, and virtual simulation software, particularly when students have to repeat difficult movements. But digital delivery is not necessarily better learning. Unedited videos, too much on-screen information, and fast visual cuts may impose processing requirements that are not needed and preventable (H'mida et al., 2020). A comparison of video vs. still images in a learning task involving a challenging skill in judo showed that continuous video was associated with superior learning performance as it maintained the continuity of perception and lessened the necessity of mental reconstruction of movement patterns (H'mida et al., 2020). The same reasoning can also be applied to Guzheng instruction: carefully planned demonstrations that provide clear correlation between hand motion, tempo, and sound might decrease the level of cognitive load, and vice versa - disorganized or incomplete material can increase it. Innovative Guzheng learning systems must thus be assessed not simply by their level of technological innovation, but rather by the degree to which they assist students to handle complexity.

### 1.3 Review scope, research gaps, and review objectives

The article falls under the category of narrative, not systematic reviews. The literature review was mainly based on articles that were written between January 2019 and 22 December 2025, although earlier literature sources were used when needed in order to form the fundamental theoretical structure of CLT. The literature reviewed concerned only literature pertaining directly to Guzheng learning, acquisition of music skills, cognitive load, multimedia teaching, and technology-supported learning, supplemented by selected literature in related areas (e.g., medical and simulation-based training) when these studies provided conceptually relevant evidence to complex procedural learning (Sweller et al., 2019; Skulmowski and Xu, 2022; Wasfy et al., 2021; Cecilio-Fernandes et al., 2020; Makransky and Petersen, 2021; Gallagher, 2023; Cole and Shields, 2019; James et al., 2020; Worschech et al., 2023; Babchenko et al., 2019; H'mida et al., 2020; Jiang and Kalyuga, 2022; Fazlollahi et al., 2022). It was prioritized to the peer-reviewed research articles that were most relevant to demonstration, segmented practice, feedback scaffolding, notation design, and digitally mediated instruction. Such a scope indicates the existing situation within the field: there is still little evidence on the subject of Guzheng, and thus a narrow, yet theoretically based synthesis is required.

Within this context as well, there are a number of significant questions that have not been addressed. The first is domain specificity. CLT has been extensively debated in the field of general education and, to a smaller extent, in connection with Western instruments, but there is relatively little research that specifically investigates cognitive load in Guzheng teaching. This is important because the Guzheng has a particular distribution of motor and expressive work between the two hands, and these characteristics can change the effectiveness of typical instructional approaches in the real world. Dividing practice into two parts (right-hand plucking and left-hand pitch control), as an example, can decrease complexity at the start of learning, but the way it influences subsequent reintegration and transfer is unknown.

The second gap is in the social organization of learning. Most of the written studies about cognitive load in music education deals with individual learners, but Guzheng training frequently takes place in groups or partial sharing environments. It brings up some practical issues of whether peer interaction can alleviate cognitive overload through the distribution of attention and problem-solving or if it adds more coordination requirements that make learning harder. Studies conducted on collaborative writing in English as a foreign language indicate that collective working memory may lessen cognitive load and enhance performance (Jiang and Kalyuga, 2022). However, that reason does not just apply to Guzheng group learning. The instrumental practice is an individual execution, and the circumstances under which collaboration becomes useful instead of being distracting have not been adequately elucidated (Jiang and Kalyuga, 2022).

Third, the cognitive load consequences of digital technologies on Guzheng education are not well-developed. New research indicates that digital devices have the potential to enhance learning if they can be created in accordance with CLT guidelines (Skulmowski and Xu, 2022), although there is limited direct data regarding specific applications to Guzheng, such as interactive notation programs, simulation systems based on virtual reality, or automated feedback tools. In yet another area, there was a randomized clinical study that revealed that an AI tutoring system had better surgical skill acquisition and transfer rates than remote expert instruction by inducing the same cognitive and emotional reactions (Fazlollahi et al., 2022). This type of result can be viewed as a possible future direction of AI-assisted Guzheng teaching, but it does not prove that technology will definitely lower the load in musical settings. Badly designed systems might also present new interfaces, distractors, or overcorrection.

A fourth gap is related to the process of learning through time. The current literature frequently describes the results of a short-term performance measure, but the acquisition of mastery in instrumental learning requires the capacity to retain it, consolidate it, and transfer it to other situations of practice. Other larger music-training experiments have shown that long-term learning has the potential to yield functional and structural gains (Worschech et al., 2023), but there is still little evidence on whether CLT-informed approaches to Guzheng training enhance retention in lasting ways or only help with short-term task performance. The difference is important since an intervention that makes things easier at the moment does not necessarily lead to robust independent performance subsequently.

Finally, the relationship between cognitive load and affective experience in Guzheng learning remains underexplored. Performance anxiety, self-consciousness, frustration, and fear of negative evaluation may compete for attentional resources and thereby contribute to extraneous load. Evidence from clinical and workplace training suggests that adverse emotional conditions may impair learning by increasing distraction and self-regulatory demands (Babchenko et al., 2019). Comparable evidence in Guzheng education remains limited. Future studies should therefore examine how motivational climate, teacher feedback style, and learners’ emotional states interact with instructional design.

**Figure 1:**
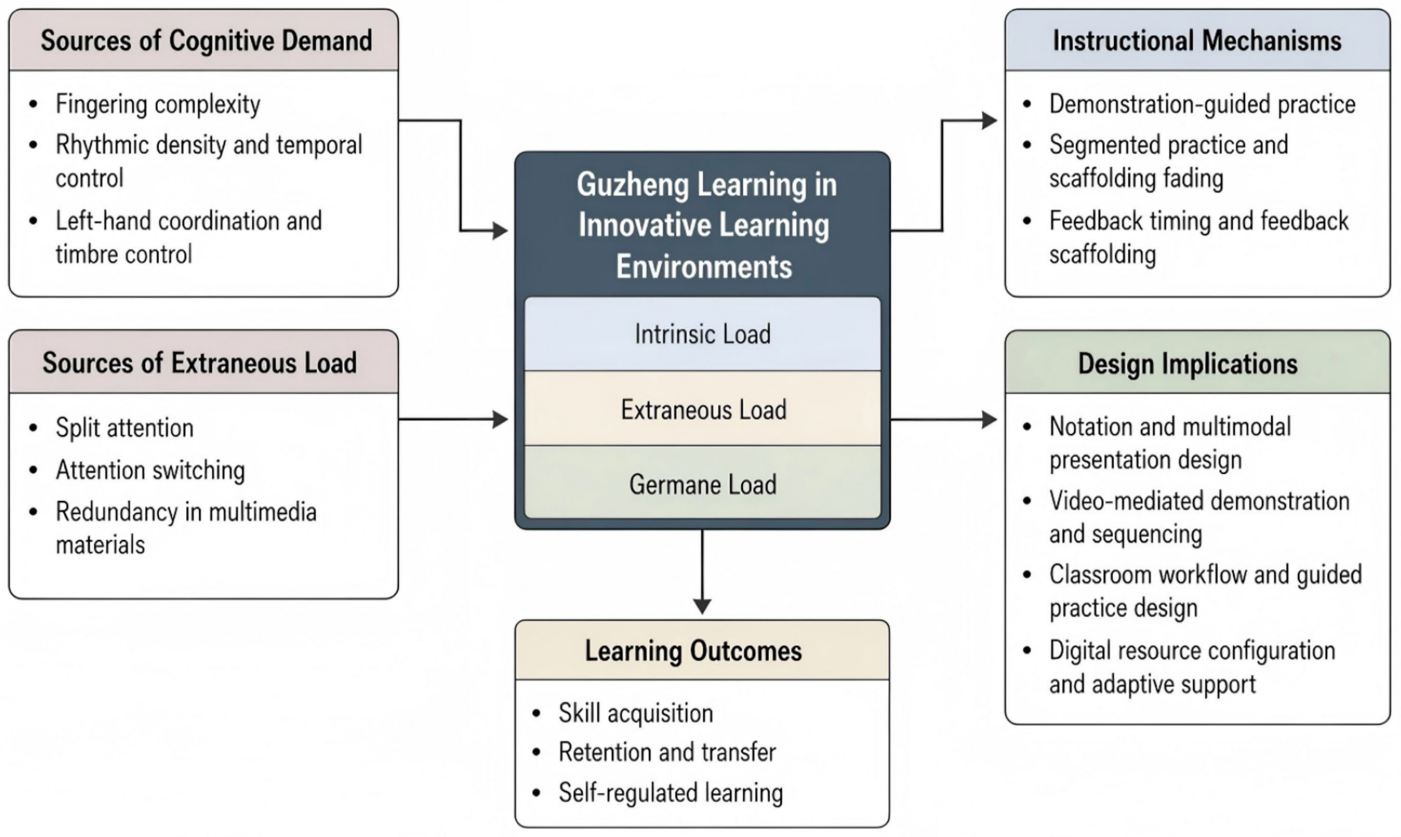
A conceptual framework for optimizing cognitive load in innovative Guzheng learning environments.

Against these gaps, the present review synthesizes evidence on cognitive load optimization in Guzheng learning, focusing on demonstration, segmented practice, and feedback scaffolding. Specifically, it aims to: (1) examine how live, video-based, expert, and peer-led demonstrations may influence cognitive load and skill acquisition; (2) evaluate whether part–whole sequencing and progressive complexity can make difficult material more manageable without weakening later integration; (3) consider how feedback timing and modality may affect cognitive load, retention, and performance refinement; and (4) identify conceptual and empirical priorities for future research. Figure 1 summarizes the conceptual framework linking cognitive demands, extraneous load, instructional mechanisms, design considerations, and learning outcomes.

The given figure represents the general conceptual framework of the review. It is focused on the analysis of Guzheng learning in innovative learning settings as the core topic and indicates the way by which the two significant input areas, namely, sources of cognitive demand and sources of extraneous load, influence the cognitive conditions of the learning process. The framework also demonstrates how instructional strategies and design implications may be employed to control intrinsic, extraneous, and germane load and thus impact upon the most important outcomes of learning, such as skill acquisition, retention, transfer, and self-regulated learning. The figure summarizes the core claim of the review that successful Guzheng pedagogy is based not only on the control over the complexity of the tasks but also on the development of learning environments, which minimize the unnecessary processing requirements and facilitate the creation of meaningful schema.

### 1.4 Methods of the review

#### 1.4.1 Data sources and search strategy

This article was designed as a narrative review. A purposeful literature search that identified literature related to cognitive load in Guzheng learning and similar areas of instrumental and music-skill acquisition was performed using two databases, namely Web of Science Core Collection and Scopus. The last revision date was 22 December 2025, and the main search window was used to select articles published after 1 January 2019 through 22 December 2025. The selected period aimed at covering the current trends in cognitive load studies, multimedia teaching, online and blended learning settings, and music education with the help of technologies. Previous ones were included selectively when they formed the basis of Cognitive Load Theory (CLT), multimedia learning, or particular instructional processes that directly pertained to this review, such as split attention, redundancy, worked examples, segmentation, and feedback timing.

The Boolean operators were applied to merge the search terms in three major concept clusters: (i) instrument and domain terms (e.g., Guzheng, zheng, Chinese zither, music learning, instrumental learning, music education, skill acquisition); (ii) cognitive framework terms (e.g., cognitive load, cognitive load theory, mental effort, and working memory); and (iii) instructional-design terms (e.g., demonstration, worked example, segmented practice, part-whole practice, feedback, scaffolding, multimedia instruction, video learning, notation design, digital learning, online learning, virtual reality, augmented reality and artificial intelligence). The representative search logic was organized in such a way that:

(“Guzheng” OR zheng OR “Chinese zither” OR “music learning” OR “instrumental learning” OR “music education” OR “skill acquisition”)

AND

(“cognitive load” OR “cognitive load theory” OR “mental effort” OR “working memory”)

AND

(demonstration OR “worked example*” OR “segmented practice” OR segmentation OR “part-whole practice” OR feedback OR scaffolding OR “multimedia instruction” OR “video learning” OR “notation design” OR “digital learning” OR “online learning” OR “virtual reality” OR “augmented reality” OR “artificial intelligence” OR AI)

The database-specific syntax has been modified appropriately on some occasions to accommodate the dissimilarity in indexing fields as well as the search interfaces.

Web of Science Core Collection has been chosen as the main initial database due to its comprehensive scope of interdisciplinary educational research and its appropriateness to be used in transparent topic-based retrieval. Scopus was also searched simultaneously to enhance the coverage in the field of education, psychology, and applied learning research. There was no hard limitation on the type of documents during the search phase, so that recall is maintained, but relevance, conceptual fit, and report adequacy were used in screening.

The initial search returned 134 records in both databases. Following the elimination of duplicates, there were 102 records left to be filtered by titles and abstracts. Out of them, 45 were kept to be assessed on the basis of full texts, and finally, 33 studies made it into the review. It was on the basis of these studies that the thematic synthesis presented below was mainly based.

Since focused research on Guzheng is still scarce, the review took into account evidence in related fields, which included general music and instrument learning, multimedia learning, simulation-based training, and other complex procedural learning environments. Nevertheless, those studies were selectively included and carefully interpreted. In this current review, evidence was given hierarchical treatment: direct studies of Guzheng learning were deemed the most relevant in context; broad music and instrument learning studies were seen as a secondary form of support; and results of adjacent non-musical domains were used as an additional tool to explain what can be transferred as the mechanism of instruction rather than as a direct proof of Guzheng education.

#### 1.4.2 Literature selection and synthesis

Once the first search had been conducted, the results of the two databases were filtered by steps depending on title, abstract, and full text (when required). The duplicate records were discarded prior to the thorough screening. A priority was assigned to those works that were explicitly relevant to at least one of the following fields: cognitive load in musical or instrumental learning, instructional design of complex skill acquisition, multimedia or video-based learning, segmented practice, feedback scaffolding, notation-related design, and technology-enhanced learning environments. When a study was irrelevant to learning or instruction, it did not have enough relevance to cognitive load or instructional design, or if it was mainly a technical report with low educational interpretability, it was excluded. Adjacent domain studies were kept, but only when they provided a mechanism-level understanding that could be reasonably transferred to Guzheng learning.

The purpose of the review was not to come up with an in-depth enumerative synthesis or a structured meta-analytical comparison since it was written as a narrative. The chosen literature was instead sorted thematically based on three focal areas of instruction, namely demonstration, segmented practice, and feedback scaffolding. The themes that were kept are because they kept repeating in the filtered literature and were relevant to the main issue of the review, which is how instructional design can control cognitive loads during complex Guzheng learning. The studies were allocated to one or more of the aforementioned themes depending on where their main focus of instruction was, and crosscutting evidence was discussed regarding cognitive demands, sources of extraneous load, mechanisms of instruction, and possible implications of Guzheng learning.

The review was not conducted as a systematic review or meta-analysis, so no formal quality scoring system has been used. Nevertheless, interpretive weight was placed on those studies that reported their methods more clearly, were more conceptually relevant, and were more directly applicable to the study of Guzheng or to other close instrumental-learning situations. It was stated explicitly in the interpretation that the conclusions were based mainly on the evidence of general music learning or other adjacent domains instead of using direct research of Guzheng in an attempt to prevent overestimation of the strength of domain-specific evidence.

## 2 Cognitive demands and sources of difficulty in Guzheng learning

Learning the guzheng is cognitively challenging, though not simplistic or unidimensional. The learning process requires the learner to integrate sensory input, motor output, and musical cognition simultaneously. Some of it is overt; some of it is covert. The degree of a task does not simply depend on its complexity on the page or in practice. Frequently, difficulty refers to the number of elements that need to be processed simultaneously, the degree to which movements need to be controlled, and the amount of attentional regulation needed in practice and execution. In the context of the Cognitive Load Theory (CLT), the issue of Guzheng learning can be explained as an unstable interaction between the technical requirements and the mental workload necessary to handle them. This part identifies three common sources of difficulty in Guzheng education, such as fingering complexity, rhythmic density and time control, and left-hand coordination with timbre control, each of which seems to impose different cognitive requirements on working memory, motor planning, and sensory integration.

### 2.1 Fingering complexity

Task difficulty is an important contributor to the complexity of fingering that is very apparent and obvious in terms of Guzheng learning. Simple plucking movements (including the use of the index finger or thumb) are even at the beginner stage of learning and are required to develop new movement patterns and start establishing stable motor routines (Casas-Ortiz et al., 2023). These motions appear to be simple to experienced performers. To novices, they are not. More complex technical movements may require concentration, deliberate action, and uninterrupted attention to the accuracy of their movements. Such a combination is sufficient to induce considerable cognitive load at the initial levels of learning.

With further instructions, it is no longer simply due to the fact that movements are getting faster, but also due to the necessity of organizing them into more and more complex sequences. Rapid flowing passages, multi-finger combinations, as well as alternating patterns demand preparation, execution, and monitoring of movement sequences at short intervals. In the CLT framework, the intrinsic load increases since there are more interactive components, which should be controlled by a finite working-memory structure (Skulmowski and Xu, 2022). It is not just a physical problem. Students need to keep a sequence in their memory, foresee the next step, and make adjustments to the new mistakes without disrupting the flow.

The requirements are even higher when the students come across longer methods that add to the expressive abilities of the instrument. Reorganization of formerly developed motor habits is common in modern-day Guzheng performance, where pitch bending, sliding, harmonics, and non-traditional plucking positions are used (Zhang and Lee, 2024). These methods rely on auditory feedback, kinesthetic sensation, and spatial control at one time. Precision in pitch bending is not just about finger pressure but also about the ability of the learner to perceive pitch change as it happens and adapt their actions to it. Such demands, if introduced earlier than necessary or when there are several other competing cues, can be quite taxing on working memory (Skulmowski and Xu, 2022).

In terms of learning, this development of fingering relies on a slow transition between controlled processing and more automatic performance (Skulmowski and Xu, 2022). At the beginning of training, students commonly observe every movement purposefully, and their work is usually slow, labor-intensive, and susceptible to failure. Through the repetition of practice, certain movement patterns will become more stable and require less conscious control. Automation is, however, not even. Some repetitive or predictable patterns may be stabilized fairly fast, whereas other non-repetitive or structurally dense passages might be cognitively demanding longer on average (Casas-Ortiz et al., 2023). It can be seen as an extension of a larger trend of motor learning studies in which it is shown that the sequential acquisition of motor skills requires a close interaction between both the cognitive and motor systems, such as the role played by the cerebellum in the learning of sequences (Ballard et al., 2019).

The instruction can make them stronger or relieve them. Online digital feedback of finger location, motion-capture, or sensor-based assistance might assist learners in identifying and fixing mistakes more effectively (Zhou and Liu, 2023). However, the effect is likely to vary according to learner characteristics and system design. When these systems provide too much visual indication or technical cues all at once, they could increase instead of decrease cognitive load (Skulmowski and Xu, 2022). Practically speaking, the complexity of fingering seems easier to handle when hard sequences are introduced gradually, divided into sections, and accompanied by feedback that focuses attention on the most important aspects of performance as opposed to everything in one go.

### 2.2 Rhythmic density and temporal control

Another important cause of difficulty in Guzheng learning is rhythm. The rhythmic density means the amount of notes or events that should be heard during a certain time frame, and the temporal control is the ability of the learner to sustain the accuracy of timing, synchronize the movements with the pulse, and vary the performance depending on the expressive purposes (Skulmowski and Xu, 2022). These requirements are cognitively important as rhythmic performance involves more than hearing a pattern or pulse. It needs to transform auditory perception into exactly timed behavior.

Relatively sparse rhythmic patterns can still be managed by novice learners since they provide sufficient time to plan and execute them. Nonetheless, with an increase in density, the processing window shrinks, and the work can prove significantly more complex than initially thought. More rapid subdivisions, more dense note groupings, and particularly in the case of polyrhythmic structures demand a higher rate of perception, greater attentional control, and finer timing of movements (Skulmowski and Xu, 2022). When two temporal organizations have to be sustained simultaneously, the pressure is increased. In these situations, learners have to inhibit interference between two rhythmic streams as well as maintain control over one, using higher-order cognitive processes like attentional switching, inhibitory regulation (Richter et al., 2024).

The concept of temporal control in the study of Guzheng is not restricted to maintaining a consistent tempo. They are also supposed to express timing dynamically by means of dynamic contrast, phrasing, and sometimes even rubato. This introduces an extra level of complexity. Expressive timing is not just mechanical precision; it relies on the learner developing the ability to understand the structure of a phrase, style conventions, and the musical intention. This type of meaning-based processing can be viewed as highly similar to germane load in CLT terms since learners do not simply respond to external timing signals but construct more elaborate musical schemas that facilitate expressive performance (Skulmowski and Xu, 2022). But expressive timing is still challenging. The border between freedom and control must be negotiated by learners over and over again.

Studies on rhythm and motor learning can assist in explaining why such demands are important. Studies have shown that the cerebellum is related to time and sequence learning, and research involving transcranial direct current stimulation indicates that neural modulation can influence the learning of sequential motor tasks (Ballard et al., 2019). Other research has shown that musical expertise correlates with increased connectivity between auditory and motor areas, which could be used to facilitate a more efficient processing of rhythm (Olszewska et al., 2021). These results do not directly correspond to the Guzheng pedagogy, and they ought not to be taken as such. Although this is the case, these findings help to make a larger point, namely that rhythmic training can be considered both cognitively and neurophysiologically, and the enhancement of temporal control is probably going to rely not solely on repetition but also on how practice is arranged and executed at what pace.

Instructionally, rhythmic complexity could be made more manageable when the timing requirements are externalized in a supportive manner. The use of metronomes, rhythmic notation, playback with guidance, and tempo-orientation digital feedback can ease the monitoring process partially by making temporal structure more apparent (Skulmowski and Xu, 2022). This kind of help can be effective if it does not exceed certain proportions. Excessive cues at the same time can transform help into interference. That is why rhythmic training can appear to be effective with segmentation, gradual tempo control, and stepwise reintegration of the more challenging passages. These techniques will not eliminate intrinsic difficulty. But they might make it less difficult to learn.

### 2.3 Left-hand coordination and timbre control

The third challenge of learning the Guzheng is the special position of the left hand. In contrast to most other instrumental scenarios where one hand plays mostly a supporting role, Guzheng playing entrusts the left hand with an extremely expressive and challenging part. Although the right hand may indicate the melody by plucking, the left hand alters pitch, vibrato, resonance, and makes a major contribution to timbral expression. Such allocation of functions necessitates the students to control various kinds of movements on different levels of music, sometimes in the same phrase (Casas-Ortiz et al., 2023). Left-hand work is thus not a superfluous ornament placed on top of the basic technique. It is already a complication to start with.

Beginners need to coordinate difficult fine motor control and sensory monitoring even to perform fairly easy left-hand maneuvers, such as pressing the string to change the pitch. The learners have to estimate the pressure, place, timing, and the pitch result as well as synchronizing these movements with the right-hand plucking. Still more advanced methods, such as playing chords or other simultaneous actions on several strings, smooth sliding moves, and regulated pitch bending across musical phrases, require even more accuracy and between-finger cooperation (Skulmowski and Xu, 2022). These constraints increase the natural load since several interacting factors have to be grouped together instead of being dealt with separately.

Timbre control is the next level. Vibrato and glissando are not just ornamental; they are essential bearers of musical character. Students will need to find out that small variations in speed, pressure, and movement influence expressive result, typically by experimenting and adjusting until finding a satisfactory solution. The procedure relies on the combination of auditory and kinesthetic feedback. It is mentally taxing, too. Not only do students have to decide if a particular method was performed, but also whether it has created the desired sound.

The challenge is even more complex when the students are required to shift rapidly between various left-hand operations in a brief paragraph. It may be necessary to make an instant shift in pitch modulation, slide action, and finally, vibrato, with all three being coordinated with the right hand in its rhythmic and melodic activities. This fast switching puts strain on cognitive flexibility and inhibition of responses, and both are connected to higher-order executive control (Richter et al., 2024). Studies on motor learning indicate that these skills are based on coordinated actions that engage prefrontal and cerebellar areas, and musical skills research indicates that motor and auditory areas undergo structural and functional changes in order to perform more efficiently (Ballard et al., 2019; Criscuolo et al., 2022). These results imply that left-hand fluency in Guzheng learning would not be achieved by repetition alone. Sequence is important. The time of providing support also counts.

The use of left-hand coordination is highly susceptible to the type of instructional support given. The scaffolded feedback could be useful during the initial stage of learning static positions and pitch changes, especially when the support is decreased incrementally with the improvement of control (Skulmowski and Xu, 2022). Technologies that provide visual support, such as augmented feedback devices, might also assist students in detecting positioning errors more effectively (Zhou and Liu, 2023). However, additional direction is not always beneficial. Too much prompting can be counterproductive when learners start to depend on external prompts rather than working on building their internal self-monitoring capability (Skulmowski and Xu, 2022). Interleaved practice could serve as one of the solutions to this issue. Switching between the left-hand methods in one practice session can enhance cognitive flexibility and learners will be able to switch between expressive requirements (Richter et al., 2024). When used with care these strategies can assist in bringing practice out of the realm of technical execution as an isolated skill and into a more integrated and musically significant form of control.

### 2.4 Implications for instructional design

Overall, fingering complexity, rhythmic density, and temporal control, and left-hand coordination with timbre regulation seem to be three significant factors to be considered when learning Guzheng. They have all contributed to task challenge through slightly different means that require various mixtures of perceptual processing, motor planning, attention regulation, and working-memory capacity. Task difficulty in Guzheng education must thus not be regarded as an immutable feature of repertoire alone. Rather, it is more appropriately viewed as a relationship between what the task demands and what the learner can at present coordinate in certain instructional conditions (Skulmowski and Xu, 2022).

This observation has a practical application. After teachers are able to identify more precisely what aspects of cognition would be problematic, they can organize practice in a more calculated manner, choose materials more attentively, and determine the times when simplifying, breaking down, or supporting performance requirements would be necessary. The aim is not to categorize the repertoire purely as a means to an end. It is to facilitate complexity without losing the artistry of the instrument. This is even more critical in digital and blended environments, where the display of information is likely to facilitate focus or distract attention. The following section then discusses three instructional mechanisms that are particularly pertinent to cognitive load management in the context of Guzheng learning, namely demonstration, segmented practice, and feedback scaffolding. Table 1 presents the key sources of cognitive demand covered in this review and the potential implications of these sources to instructional design in Guzheng learning. The summary is based on the multi-layered evidence base incorporating all directly Guzheng-relevant evidence when present, general music and instrumental-learning literature, and some adjacent-domain studies as interpretational support.

**Table 1:** Sources of cognitive demand potentially relevant to Guzheng learning.

Source	Illustrative Guzheng manifestation	Potential cognitive implication	Possible instructional consideration	References
Fingering complexity	Single-finger plucks, multi-finger combinations, rapid sequences, and extended techniques	May impose substantial intrinsic load through movement sequencing, motor planning, and continuous performance monitoring	Enter patterns gradually; break down harder parts; provide selective examples and feedback to guide focus on the most important details	Skulmowski and Xu (2022); Casas-Ortiz et al. (2023); Zhang and Lee (2024); Ballard et al. (2019); Zhou and Liu (2023)
Rhythmic density and temporal control	Fast subdivisions, changing tempo, polyrhythms, and expressive timing	May increase working-memory demands associated with auditory-motor coordination, attentional control, and temporal regulation	Use gradual tempo progression, rhythm-focused segmentation, and forms of guided metrical support where appropriate	Skulmowski and Xu (2022); Ballard et al. (2019); Richter et al. (2024); Olszewska et al. (2021)
Left-hand coordination and timbre control	Pressing, sliding, vibrato, glissando, and rapid switching among expressive actions	May place high demands on kinesthetic monitoring, auditory judgment, and cognitive flexibility	The ordering of left-hand skills should be in terms of stability or dynamic nature of control; integration with the right-hand movements should be scaffolded	Skulmowski and Xu (2022); Casas-Ortiz et al. (2023); Ballard et al. (2019); Richter et al. (2024); Criscuolo et al. (2022)
Multi-component integration	Combining fingering, rhythm, pitch shaping, and expression within the same passage	High element interactivity may increase overall intrinsic load, especially during early learning	Do not integrate completely until partial control is achieved; reattach parts by means of directed whole-task rehearsal	Skulmowski and Xu (2022); Casas-Ortiz et al. (2023); Richter et al. (2024)

This table is a summary of the sources of cognitive demand that were mentioned in the review as possible sources in the context of Guzheng learning. The consequences mentioned here should be read with caution. A number of entries have been based on research directly related to the study of Guzheng, but there are other entries based on more general studies on music learning and motor learning. When it comes to situations where direct Guzheng-specific evidence is still scarce, the table represents the interpretation at the level of mechanisms instead of confirmation at the level of domains.

## 3 Sources of extraneous load in Guzheng instruction

The combination of conventional teaching methods with online resources has broadened the opportunities in Guzheng education. It has also complicated the process of designing instructions. According to the Cognitive Load Theory (CLT), learning is more inefficient as mental resources are used up to process data that adds little value to schema creation or the development of skills (Sweller et al., 2019; Skulmowski and Xu, 2022). This concern is particularly problematic in Guzheng instruction since students frequently have to coordinate notation, hand movements, audio feedback, and expressive control with restricted working-memory constraints. If the materials or routines are disorganized, a significant portion of the cognitive energy could be used up in organizing the formats of presentations instead of learning the skill.

In this review, there are three sources of extraneous load that seem to be particularly relevant: split attention between notation and fingering annotations, attention switching during demonstrations, and redundancy of audio-visual and textual materials. Such issues are not peculiar to the study of the Guzheng. However, they can be especially significant in this case since instrumental training is very sensitive to the precise alignment between perception and action. Minor issues in design may turn out to be more practically significant than they initially appear to be.

### 3.1 Split attention between notation and fingering annotations

One popular source of extraneous load in Guzheng learning occurs when the pitch information and fingering instructions are provided in discrete form. Most notation systems, either number-based or staff-based with added symbols, require learners to constantly shift back and forth between the primary score and another set of signals representing fingering, hand position, or expressive technique (Mirza et al., 2019). In the view of CLT, this setup is problematic as it requires learners to first mentally combine separate sources of information and then act on the combined information (Sweller et al., 2019). Such integration is not costless. It uses up working-memory capacity that could have been used to help perform control or schema formation.

The issue manifests itself in practice when students are shifting their focus back and forth between the melodic line and the fingering symbols that appear on another part of the page. A beginner playing a work like High Mountain and Flowing Water may have to not only recognize pitch patterns, but also figure out what plucking style or left-hand change goes with each section. The separation of this data in space can cause the practice to be visually disjointed. Rather than focusing on the timing, posture, and sound production, the learner wastes time cross-checking cues. The problem that looks like a notation problem is in fact a cognitive-load problem.

The identical challenge could also be intensified in digital media. A learner may be requested to view a demonstration video in one window of an online tutorial or an app-based practice environment and in another, read a score. Studies on digital and online learning indicate that the split-attention presentation may enhance extraneous load in case information, which should be combined, is divided into poorly coordinated sources (Skulmowski and Xu, 2022). The problem with Guzheng instruction is not that the notation, fingering, and demonstration are absent, but they can be present in such types that do not interact well.

Previous experience will probably influence the intensity of this phenomenon. More mature schemas can help students make up for the difference in split presentation because they have already learned to associate notation and movement. Beginners tend to be less safe. The mappings are not yet stable in them, which means that dividing attention might be more expensive (Trypke et al., 2023). One study on split-attention and redundancy effects came to the same conclusion: combined materials may be more effective with lower levels of previous knowledge learners (Trypke et al., 2023). This implies that fingering symbols and technical cues can be more useful in Guzheng education when they are placed as closely as possible to the appropriate section of the notation, rather than being distributed across different layers.

### 3.2 Attention switching during demonstrations

The demonstration is still at the core of the Guzheng learning process. Traditional and digital learners rely significantly on observing the expert movement, hearing the sound quality, and listening to the verbal explanation. But demonstrations are not always effective since they are information-rich. They might also put a heavy extraneous load when learners need to constantly shift their attention between hand movements, spoken explanation, score information, and auditory output (Elchlepp et al., 2021).

In the usual classroom setting, an instructor could be showing a right-hand technique as they talk about rotating wrists and then switch rapidly into a left-hand movement that alters pitch or tone. Novice learners might need to reorient themselves many times. A decision must be made as to what is seen, heard, and remembered, frequently in a very short time. Attention switching studies indicate that these switches cost cognitive resources since the learner has to disengage one task configuration and reorganize it to another (Elchlepp et al., 2021). The switching process itself can decrease processing efficiency even if all the information is relevant.

It is especially critical in learning Guzheng since most of the methods require fine-tuned timing interactions between the two hands. When a student pays more attention to the right-hand plucking of the teacher, the action of the left hand that changes the pitch might not be noticed or comprehended fully. Studies on attentional repulsion have demonstrated that sudden changes in visual attention may change the perceived time or location of objects, particularly when there is more than one visual attribute being monitored simultaneously (Baumeler et al., 2020). It is possible that in instrumental training, such distortions might actually add to erroneous imitation, but specific Guzheng evidence is not yet substantial.

The use of a digital teaching environment can worsen the situation. The teacher, with the help of a webcam, digital score display, audio playback, and slide-based explanation, may easily establish a tight multimodal environment where the students have to select between conflicting information streams. Studies on digital learning environments indicate that the level of cognitive load is likely to increase whenever more than two sources are offered simultaneously and there is no defined order or prioritization (Skulmowski and Xu, 2022). The problem is thus not the ineffectiveness of multimedia demonstration by itself. Rather, it is that demonstration becomes more difficult to keep up with when there are too many signals simultaneously competing.

The modality effect introduces an additional level of complexity. In other cases, visual demonstration coupled with auditory explanation has been found to ease the burden through the distribution of information across the channels (Haavisto et al., 2023). However, this advantage is conditional on coordination. Learners who would need to split their attention between oral explanation and non-related or badly coordinated visual content may not benefit as expected. There is more to it than just adding narration. When the timing of a demonstration is controlled in Guzheng tutorials, it may be more helpful if the demonstration is selective and does not require excessive switching.

### 3.3 Redundancy in audio-visual and textual materials

Another source of extraneous load on Guzheng teaching is redundancy. Redundancy can be defined in CLT studies as the case when learners are instructed to learn the same or similar information, which does not add much meaningful value to the learning (Trypke et al., 2023). It is particularly problematic in online instruction, where text, narration, graphics, notation, and video might be readily added to the same learning sequence. Although these additions are usually meant to be helpful, they can actually raise processing requirements by forcing learners to read the same message in various formats (Sweller et al., 2019; Trypke et al., 2023).

Redundancy is frequently observed in Guzheng tutorials where a teacher repeats what they say verbally as on-screen text at the same time as demonstrating the same technique in video. An example of a plucking technique lesson may have a recorded demonstration, spoken explanation, and written instructions that contain almost the same information (Kalyuga Slava et al., 2004). Certain redundancy can be useful to beginners in certain circumstances, especially if it elucidates hard material. Yet more comprehensive data indicate that the effect depends on the context. The analysis of redundancy research revealed that a positive impact would be more probable in case both learners were not experienced, and additional information would aid comprehension instead of merely repeating it mechanically (Trypke et al., 2023).

When redundancy loads the same working memory channel, the problem becomes more acute. The addition of written text to a visually intensive demonstration could be crowding the visual channel at exactly the time when learners are attempting to follow finger movement, string position, and time (Trypke et al., 2023). A study of redundant audio-visual learning materials using EEG revealed higher levels of cognitive load in redundant conditions and thus indicated that learners were using more cognitive resources to process the duplicated material (Bayraktar et al., 2024). When learning Guzheng, such an overload may result in mental fatigue that causes decreased attention to the movement pattern that is being learned.

Redundancy is especially hard to control in mobile or app-based learning settings. Scrolling scores, instructor videos, text descriptions, pop-up tips, and reminder notes are examples of the digital tools that have some overlap with each other and frequently repeat some content (Skulmowski and Xu, 2022; Curum Brita and Khedo Kavi Kumar, 2021). Studies on mobile learning and cognitive-load management indicate that this repetition can turn unproductive when the design is not prioritized clearly (Curum Brita and Khedo Kavi Kumar, 2021). This might happen in Guzheng apps where the learners experience repeated reminders, duplicate explanations, or overlapping displays, all of which are supposed to facilitate the practice but together make the work more difficult to handle.

A further problem is that learners do not necessarily identify redundancy by themselves. According to studies on cognitive load self-management, numerous students go on to process redundant information instead of selectively ignoring it when it is no longer needed (Mirza et al., 2019). When practicing the Guzheng, a student can keep viewing a video, reading the textual explanation, and listening to the narration even when the main movement is already comprehended. Redundancy cannot be eradicated as an ongoing impediment to successful practice unless learners are assisted in distinguishing between what is important and what is not.

Redundancy effects may also depend on emotional state and well-being. A survey of well-being and cognitive load has proposed that positive emotional functioning might also act as an overload buffer by enhancing attentional regulation (Hawthorne et al., 2019). Implications of this are that students undergoing stress or performance anxiety will struggle to eliminate non-essential repetitive information in course materials. The properly developed Guzheng materials must not rely on an assumed perfect control of attention. The redundancy should be addressed during the instructional design rather than being fully dependent on the self-regulation of the learners.

### 3.4 Implications for Guzheng learning environments

All in all, split attention, attention switching, and redundancy seem to be the three critical sources of extraneous load in Guzheng instruction. Their functioning is not similar, and the power of direct Guzheng-specific evidence does not have uniformity among them. However, even all three may redirect cognitive resources to the detriment of the construction and perfection of musical skills.

The overall message is quite straightforward: additional resources are not always a guarantee of improved learning. Cues on notation and fingerings should be more appropriately synchronized; demonstrations must be less ambiguous and with fewer conflicting signals; and multimedia content should not have redundant or uncoordinated channels of information. These are not just technical improvements. They determine if learners will be able to concentrate on time, movement, and expression, or will be forced to manage the format. The next section discusses instructional mechanisms that may help regulate these demands more actively during Guzheng learning. Table 2 summarizes the major sources of extraneous cognitive load discussed in relation to Guzheng instruction. Its entries draw on direct Guzheng-related discussion where available and on broader cognitive load and digital-learning research for mechanism-level interpretation.

**Table 2:** Sources of extraneous cognitive load potentially relevant to Guzheng instruction.

Source	Illustrative Guzheng manifestation	Potential cognitive implication	Possible design consideration	References
Split attention	Notation, fingering, and expressive cues presented separately; score and demonstration shown in disconnected formats	Students might have to mentally combine different sources of information, which could add unnecessary processing loads	Put the notation and fingerings closer together; match up the score display with the most applicable demonstration sections as appropriate	Sweller et al. (2019); Skulmowski and Xu (2022); Mirza et al. (2019); Trypke et al. (2023)
Attention switching	Learners alternate among teacher movement, verbal explanation, notation, and sound	Repetitive switching can lower the efficiency of processing and make delicate perceptual coordination harder, in particular, when dealing with novices	Sequence demonstrations more selectively; avoid concurrent overload from multiple competing streams	Skulmowski and Xu (2022); Elchlepp et al. (2021); Baumeler et al. (2020); Haavisto et al. (2023)
Redundancy	Similar information repeated across video, narration, text, and annotation	Duplicate processing may increase unnecessary load, particularly when the same channel is repeatedly burdened	Reduce non-essential repetition; avoid text-heavy overlays during visually demanding demonstrations	Sweller et al. (2019); Mirza et al. (2019); Trypke et al. (2023); Kalyuga Slava et al. (2004); Bayraktar et al. (2024); Curum Brita and Khedo Kavi Kumar (2021); Wan et al. (2023)
Poor digital coordination	Multiple windows, excessive prompts, dense overlays, or unnecessarily complex interfaces	Interface management may compete with musical learning and weaken attentional focus	Design for clarity, restraint, and better coordination of information flow across digital resources	Skulmowski and Xu (2022); Curum Brita and Khedo Kavi Kumar (2021); Hawthorne et al. (2019)

This table summarizes sources of extraneous cognitive load that may be relevant to Guzheng instruction. The implications should be interpreted cautiously. Several entries are supported mainly by broader cognitive load and digital-learning research rather than direct Guzheng-specific evidence. The design considerations are therefore theoretically informed and require domain-specific validation.

## 4 Instructional mechanisms for cognitive load optimization

If the extra load on Guzheng education is mainly due to preventable design issues, then good teaching should not just make the information understandable but also arrange learning so that learning of skills that are complex can be achieved. In literature, the three mechanisms that have been most cited are worked examples through demonstration-guided practice, segmented practice with fading scaffolds, and feedback that is calibrated in timing and intensity. Such mechanisms are not the same, and they are not necessarily as directly supported by evidence in Guzheng settings. Nonetheless, when combined, they provide a valuable starting point in considering how unproductive processing demands could be minimized, and support for schema formation, performance refinement, and self-regulated learning would slowly be provided.

### 4.1 Worked examples and demonstration-guided practice

Worked examples allow learners to have access to a successful performance model prior to engaging in independent work. It could be in the form of teacher demonstration, annotated videos, or gradual teaching in Guzheng learning, whereby the learner would initially observe, then imitate with assistance, and finally do it on their own with more freedom. They probably have cognitive utility because they minimize the necessity of novices to come up with answers, yet they still attempt to comprehend the problem (Nückles et al., 2020). It is important in the initial stages. This support may be especially useful when novices would otherwise need to solve several unfamiliar movement problems simultaneously.

It is particularly important in starting Guzheng lessons where students are required to simultaneously juggle several novel aspects such as finger positioning, order of movements, posture, and timing. Demonstration can be used to steady attention as it makes the task structure more noticeable. Studies on scaffolding in collaborative and technology-assisted learning indicate that outside assistance is usually more useful if it is adapted to the experience of learners instead of being provided in the same manner (Tammeleht et al., 2020; Janson et al., 2019). When it comes to teaching Guzheng, it means that novices might be able to gain advantage of explicit demonstrations with slow modeling and short explanations, whereas advanced learners might need less overt direction and space to make interpretative judgment.

Worked examples can also be used to facilitate self-regulated learning in conjunction with reflection. A larger body of research on instructional support and self-regulation indicates that learners would profit when they were prompted to track where they had difficulty and what parts of a model they were able to recreate successfully (Nückles et al., 2020). The repetition is not substituted in Guzheng practice. It alters the nature of repetition. Reflection might assist in shifting the focus of the effort on the more unstable aspects of performance rather than motivating students to play the same passage over again without any evaluation.

The technology-enhanced scaffolds also have the potential to provide some of these advantages as long as they are used with moderation. Augmented reality applications can be used, for example, to assist students with tracking the position of their fingers or the path of their movements by adding visual cues on top of the instrument or its digital form (Singh et al., 2019; Singh et al., 2020). There are tools that do not require changing the performance space of an instructor and one’s own frequently. However, the gain is bound. When the interface is visually cluttered, too directive, or too novel, it can create a new level of extraneous load instead of eliminating one. This caution has a larger application. Demonstration must not be so directive that it takes away all learner agency. Research based on CAMIL also indicates that agency and engagement have a continued role in contexts rich in technology (Makransky and Petersen, 2021). That is why demonstration-guided practice seems to be most effective when it progressively moves out of explicit modeling, into more responsibility of the learner, than leaving learners in a state of eternal dependence.

### 4.2 Segmented practice and scaffolding fading

Segmented practice manages intrinsic complexity by dividing a difficult passage into smaller units defined by phrase, hand function, technical problem, or tempo. In Guzheng learning, a student may first stabilize a right-hand sequence, a left-hand pitch-shaping action, or a rhythmic pattern before combining these components. This approach temporarily reduces the number of interacting elements during early practice without changing the complexity of the final performance task.

The effectiveness of segmentation depends on how instructional support is withdrawn. Beginners may require explicit prompts, slowed practice, and clear task boundaries, whereas more experienced learners may benefit from less directive guidance (Tammeleht et al., 2020). Support should therefore decrease as performance becomes more stable. In practice, early work may involve guided repetition of individual components, followed by increasingly independent organization and combination of those components.

Self-monitoring can support this transition. Checklists, brief self-assessment prompts, or performance rubrics may direct attention to the most important feature of each segment (Nückles et al., 2020; Nooijen et al., 2024). These supports should be simplified or removed as learners become more capable of identifying errors independently. Adaptive systems may also adjust tempo, prompts, or demonstrations in response to recurring errors, although direct evidence from Guzheng learning remains limited (Li et al., 2023; Azevedo et al., 2022).

Segmentation also has a clear limitation. Excessive division or delayed reintegration may prevent learners from developing a coherent representation of the complete passage. Narrow practice does not automatically produce flexible transfer (Sala et al., 2019). Segmented practice should therefore be followed by guided whole-task rehearsal so that technical components are reconnected within musically meaningful performance.

### 4.3 Feedback timing and feedback scaffolding

In the case of instrumental learning, feedback cannot be avoided, but its usefulness does not depend solely on its content. The timing is also important. Feedback in Guzheng teaching may focus on hand position, control of force, pitch, timing and expressive shaping, and all these dimensions are not best suited to the same timing or frequency of intervention. Instantaneous feedback can assist in stopping the repetitive motor mistakes early before they become habitual. Delayed feedback can provide more space to evaluate oneself and process larger performance patterns more deeply. Both approaches may be useful, depending on the learner, task, and stage of practice.

To beginners, the prompt response might be especially critical when there are still unstable motor routines that form the basis. Early intervention could help to prevent a pattern of ineffective hand positioning or wrong plucking strength if the learner were to use them again. But immediacy is no assurance of quality. Excessive frequency of feedback, its density, or the fact that it is interruptive can cause learners to become dependent on corrective feedback of others and lose fluency as they process further instructions. Studies have indicated that accuracy of feedback is frequently more important than timing alone, however, timing does influence the amount of cognitive load that is experienced at the time (Brand et al., 2020).

The role of delayed feedback is slightly different. It leaves space to reflect and self-monitor by letting the learner finish an attempt prior to the comment. Such a change might make a difference. Recording the performance and then watching it again subsequently either individually or with a teacher might assist in identifying recurrent issues that are more difficult to observe when performing live (Nückles et al., 2020). Delayed feedback would also have great utility in Guzheng practice especially when dealing with larger problems like phrasing, continuity or expressive balance since instant interruption may ruin the overall music structure.

Adaptive feedback provides a more elastic middle ground. The study of customized and technologically assisted learning settings indicates that feedback can be more useful when the feedback is responsive to the needs of the learners as opposed to being done in a fixed schedule (Fontaine et al., 2019; Yin et al., 2024). In Guzheng education, it could imply instant feedback on repetitive lower-level motor mistakes, but later commentary on higher-order interpretative or expressive mistakes after the student has finished a phrase or passage. To put it simply, timing is the solution to the problem.

Careful management of social and multimodal feedback can also be supportive in learning. Online collaborative environments incorporating personalized feedback have been linked to decreased mental load and enhanced learning results (Lanqin et al., 2021). Peer review of recorded performances in Guzheng contexts could promote reflection as well as decentralize some evaluation demands. Likewise, multimodal feedback, which may contain visual, auditory, or haptic cues, may decrease the load on a particular channel, assuming that the cues are organized instead of superfluous (Divekar et al., 2021). In fact that more feedback is not always better. There are times when it is.

Like any other type of scaffolding, feedback timing needs to depend on learner knowledge. Advanced learners might have gained more advantage through delayed or less intrusive feedback, which would allow them to solve problems independently and self-monitor, whereas novices usually require more direct assistance to build adequate technique (Tammeleht et al., 2020). That is why the most effective question in Guzheng education probably will not be whether immediate feedback is preferable to delayed feedback, or vice versa. It is more efficient to consider feedback as a scaffold that may be made more intensive, less intensive, delayed, or redistributed based on the learner, the task, and the stage of practice.

### 4.4 Implications for instructional design

Worked examples, segmented practice, and feedback scaffolding address different aspects of cognitive demand. Worked examples may reduce unnecessary problem generation during early learning. Segmentation may limit the number of interacting elements processed at one time, while calibrated feedback may guide correction without creating excessive dependence. Their value depends on sequencing: explicit support is most useful when it is gradually reduced as learners develop more stable schemas and stronger self-monitoring.

In digital or blended instruction, the same principles remain applicable. Technology may support observation, repetition, and adaptive feedback, but it may also introduce additional visual or interface demands. Instruction should therefore be organized around the learner’s current task rather than around the number of available features. The next section considers how cognitive load and learning outcomes can be measured, including retention and transfer beyond immediate performance (see Table 3).

**Table 3:** Instructional mechanisms potentially relevant to cognitive load management in Guzheng learning.

Mechanism	Main instructional role	Potential cognitive implication	Typical Guzheng application	References
Worked examples and demonstration-guided practice	Provide an external model before independent execution	May reduce unnecessary problem generation and help stabilize early attention, especially for novice learners	Teacher- or video-based modeling of fingering, posture, and timing before learner imitation	Makransky and Petersen (2021); Nückles et al. (2020); Tammeleht et al. (2020); Janson et al. (2019); Singh et al. (2019); Singh et al. (2020)
Segmented practice	Divide a complex task into more manageable units	May reduce momentary processing burden by limiting the number of elements handled simultaneously	Practicing a phrase, hand function, or technical pattern separately before later reintegration	Nückles et al. (2020); Tammeleht et al. (2020); Nooijen et al. (2024); Li et al. (2023); Huang et al. (2021); Sala et al. (2019)
Scaffolding fading	Gradually withdraw instructional support as performance stabilizes	May support a shift from externally guided practice toward greater self-regulated control	Moving from full cueing to limited prompts and then to more independent rehearsal	Nückles et al. (2020); Tammeleht et al. (2020); Nooijen et al. (2024); Li et al. (2023)
Immediate feedback	Address unstable motor errors during early execution	May interrupt inefficient technique formation, although excessive use can increase momentary load or dependence	Real-time correction of finger angle, plucking force, or hand position	Brand et al. (2020); Fontaine et al. (2019); Yin et al. (2024); Divekar et al. (2021)
Delayed feedback	Create space for reflection and self-monitoring after task completion	May support a deeper review of performance patterns without interrupting performance flow	Reviewing recordings after a full phrase or practice cycle	Nückles et al. (2020); Tammeleht et al. (2020); Brand et al. (2020); Lanqin et al. (2021)
Adaptive feedback and support	Adjust guidance according to learner needs and task difficulty	May help keep challenge and support in a workable balance under some conditions	AI- or platform-based prompts that isolate recurring errors or adjust task difficulty	Azevedo et al. (2022); Fontaine et al. (2019); Yin et al. (2024); Lanqin et al. (2021); Divekar et al. (2021)

This table is a summary of the main instructional mechanisms addressed in the review, such as worked examples, segmented practice, scaffolding fading, and feedback timing. The cognitive implications presented here have been backed up with evidence of different levels of directness. Some are based on direct discussions about Guzheng, others on more general studies of music and instrument learning, and others on related complex-skill literature that can elucidate mechanisms that may be transferable. All these entries must thus be seen as an organized form of synthesis of the existing evidence and not as a set of standard findings concerning Guzheng teaching.

## 5 Measuring cognitive load and evaluating learning outcomes

Cognitive load should be used as a meaningful basis of Guzheng pedagogy only when it is associated with reliable measures of the cognitive experience of the learners and the results that are the consequence of such experience. This is not easy. Motor coordination, auditory monitoring, visual processing, and expressive interpretation happen simultaneously during the Guzheng learning process, and there is no one indicator that will sufficiently capture these processes. A scale is not sufficient by itself. Neither is a biometric signal. The most effective approach in this field is consequently probably going to be designs that incorporate subjective reports, process data, and performance-based outcomes rather than using either source separately.

### 5.1 Subjective cognitive load scales and their validity

Despite their limitations, subjective self-report scales continue to be popular in the field due to their practicality, flexibility, and ability to measure the perceived mental effort associated with performance or post-performance. They have been found particularly effective in Guzheng studies in areas of learning where direct observation cannot be made, such as perceived difficulty, mental effort, and whether the learner perceives the task as easy or difficult. But that usefulness is conditional upon some careful adaptation. An easy-to-administer scale does not necessarily mean that the generic scale will become valid.

#### 5.1.1 Unidimensional and multidimensional scales

The conventional cognitive load studies have commonly used unidimensional measures to assess the mental effort, like the Paas scale. They may be helpful when measuring the total perceived effort, but when it is necessary to differentiate between intrinsic, extraneous, and germane load (Brondfield et al., 2021), they become less useful. The difference is relevant in Guzheng learning. The difficulty can be due to the technique itself, the design of the instruction, or the effort spent in greater depth of musical understanding. One global score cannot really distinguish those sources accurately.

Therefore, multidimensional scales are more encouraging. The Cognitive Load Scale created by Leppink et al. is especially useful since it operationalizes the three major elements of CLT into distinct groups of items (Choi and Lee, 2022). Studies conducted within the context of e-learning indicate that the scale has a meaningful factor structure and good reliability, as well as significant correlations with performance (Choi and Lee, 2022). One possible example of such an instrument in Guzheng education might be adapted to relate more specifically to finger coordination, multimodal practice requirements, or clarity of instruction. Such a value would be found there: it would not be measured in terms of load as an abstract concept, but rather it would be clarified what kind of load changes and why.

The same can be said about scales that were created to be used in modern learning conditions. The MCLS-POL, as an instance, distinguishes between noise-related, media-related, and device-related external sources of load (Andersen and Makransky, 2021). It might be important to consider the use of Guzheng in a digital or hybrid context, where ease of access and other challenges could be presented not by the musical challenge itself, but by the small screens, poor quality audio, misaligned video, or interface clutter. Here, the learner does not just have a hard time with the instrument. The environment can be a factor of the problem.

#### 5.1.2 Validity in complex skill contexts

In order to be helpful in Guzheng studies, the validity of subjective scales should be taken into consideration. The content validity is particularly crucial since general cognitive load items are not necessarily well correlated with instrumental learning tasks. The fact that measures developed in other areas, including the Consult Cognitive Load Instrument in a medical consultation context, demonstrate a more extensive point that items should reflect the real requirements of the task at hand can be illustrated by them (Brondfield et al., 2021). In this case, it would require language that focuses on coordination, auditory judgment, timing, and expressive control, instead of depending exclusively on abstract workload language to develop a Guzheng-specific instrument.

The construct validity is also important. A number of multidimensional instruments have been tested through confirmatory factor analysis, and the existing literature indicates that intrinsic, extraneous, and germane load can be measured empirically in favorable conditions (Andersen and Makransky, 2020). In Guzheng learning settings, particularly when it comes to simulated or VR-mediated interaction, these divisions might be helpful in practice since they might assist in determining if students are finding tasks too complex, instruction design not clear, or the effort involved in dealing with the environment (Andersen and Makransky, 2020).

The predictive validity is also important. The scores of a scale are significantly more informative when they can be interpreted in relation to future performance, retention, or transfer. In high-fidelity skills training, high intrinsic load has been related to poorer performance on challenging tasks, while high germane load has been related to enhanced performance on these tasks at times (Greer et al., 2022). Similar studies have not yet been conducted in the field of Guzheng, although it would be highly useful to support any argument about the effectiveness of a particular instruction design on the grounds that it is believed to be.

#### 5.1.3 Adaptation challenges in Guzheng research

Although subjective scales are useful, they are not easy to implement in the context of Guzheng studies. One persistent problem is that extraneous-load items tend to exhibit poorer reliability than intrinsic- or germane-load items, in particular when scales have been shifted across domains without adequate modification (Thees et al., 2021). It is not unexpected. The extraneous load will depend on the context. During the process of learning Guzheng, it can be due to the arrangement of notes, camera position, audio delay, screen arrangement, or the explanation style of the teacher. Such a generic item, like the instruction, was unclear and might not have enough specificity to reliably identify those differences.

The individual differences also pose another problem. The past musical experience, the choice of learning, and the transfer of instruments may all influence the interpretation of the same task by the students (Choi and Lee, 2022; Huang et al., 2019; Çakıroğlu et al., 2019). A person who has played other types of plucked string instruments before will find it much easier to play the same piece with a lower level of right-hand technique than a beginner. In such situations, it is hard to understand the scale data unless they are supplemented with information on the background of the learners. It is relevant to consider the musical experience. The same goes for the acquaintance with the learning environment.

Another required step is linguistic and cultural adaptation. Translation instruments used in non-Western environments are typically not only converted directly into the target language but must be conceptually tailored to match the local learning processes and the vocabulary of the discipline (Yıldız and Özkök, 2025). Scales used in the study of Guzheng must thus be administered in Chinese using terms that are significant in the teaching and music culture of the instrument as opposed to simply reiterating general educational language.

#### 5.1.4 Strengthening subjective measures through triangulation

Subjective scales are more convincing when they are read with the objective indicators. Physiological and behavioral measures may be used to determine if the effort reported is consistent with a change in attention or arousal. Complex learning tasks studies have indicated correlations between self-reported effort and heart rate variability, indicating that subjective experience and physiological load are not totally independent (Minkley et al., 2021). The convergence of these phenomena in Guzheng studies will not do away with interpretative ambiguity but will make self-reported results more reliable.

Eye-tracking provides a valuable addition as well. Studies of multimedia learning have demonstrated that people who report high extraneous loads tend to exhibit scanning patterns that resemble split attention, such as repeatedly switching between different sources of information (Kastaun et al., 2021). With the help of eye-tracking, it may be easier to understand in Guzheng research if students who report a lack of understanding of notation are actually splitting their attention inefficiently among score, hand position, and demonstration. Effort levels during more cognitively demanding parts of practice might also be crudely measured by the pupillary response (Mitre-Hernandez et al., 2021). Each of these measures alone cannot be considered to be definitive. All combined together, they may become less vulnerable to interpretation.

### 5.2 Process data, practice logs, and learning analytics

While subjective measures reflect the way learners perceive a task, process measures can reveal what they actually do during the time. The difference is relevant to Guzheng learning as the skill acquisition process happens through repeated practice, not during one performance instance. The duration of practice, repetition patterns, error trajectories, replay behavior, and relationships with digital tools might all point to how learners deal with complexity and at which points the difficulty shows up in action.

#### 5.2.1 Self-reported and automated practice logs

The practice logs have been in existence to record the learning activities. They have traditionally depended on the personal narratives of the learners about what they did, how long they did it, what problems they faced, and how difficult the session was (Mayer, 2018). These types of records may shed light on the ways of self-regulation, and provide visibility into the learner’s view of the progress. Its shortcomings are also clear: recall bias, inconsistency in details, and poor compliance may lower the reliability, especially in young or inexperienced learners (Skulmowski and Xu, 2022).

Automated logging presents an alternative way. Using sensor-based devices, app-assisted training devices, or digital devices, it is now able to capture repetition count, timing consistency, mistakes in finger movements, as well as a history of interactions directly (Choi and Kim, 2021). Learning Guzheng, these statistics can be useful to determine if a challenging section of the piece improves after repeated practice, if there are certain recurring technical issues, or how frequently students go back to specific demonstrations. The records provide a more sustained and unselected picture of practice behavior compared to self-report.

This is also the case with digital platforms that do not have specialized tools. Learning management systems or music-learning platforms may demonstrate the frequency of video replays, pauses on crucial parts, revisiting challenging passages, or shifting between sources (Skulmowski and Xu, 2022; Montgomery et al., 2019). These patterns cannot be used to learn alone, but they could indicate whether learners employ the materials strategically or merely struggle to understand the complexity.

#### 5.2.2 Learning analytics and pattern detection

After being gathered, processed data can be used to find wider trends of behavior among learners. In a descriptive sense, analytics can indicate the distribution of time spent on right-hand exercise compared with left-hand practice, the frequency with which learners repeat a technically difficult passage, or which teaching materials are used the most often (Choi and Kim, 2021; Montgomery et al., 2019). At a higher predictive level, the same information could be used to determine when a learner will probably plateau or need more support.

It is highly beneficial in case the aim is to figure out potential causes of cognitive load. Recent research in the field of learning analytics has demonstrated that behavioral footprints may be utilized to deduce trends associated with cognitive load within e-learning settings (Choi and Kim, 2021). Analogous analyses in Guzheng learning might also assist in establishing if learners are experiencing difficulties mainly due to notation complexity, fragmented materials, poor sequencing, or repeated failure of a particular method. These results would have made the analysis of cognitive load more practical as they relate to theory and observable learning behavior as opposed to merely theoretical interpretation.

The duration and schedule of work also count. Broader studies on the acquisition of skills indicate that spaced practice is likely to be superior to massed rehearsal in part because it minimizes prolonged overload and promotes consolidation (DeKeyser, 2020). In Guzheng education, process data might thus have been used to determine whether shorter but more regular sessions are correlated with less reported load, lower error rates, or better retention over time. It would be a more informative question than merely inquiring about the amount of time that students spent practicing.

#### 5.2.3 Interpreting process data through a CLT lens

Process data is most valuable when theoretically interpreted as opposed to simply being described. According to CLT, extended completion time, repeated mistakes, too much replay, or inconsistent practice rhythms can be a sign of not only general difficulty but also more specific issues concerning intrinsic or extraneous load. Breakdown on a repeated basis in a section with rich fingering demands may indicate intrinsic complexity, whereas repeated pausing due to score-video synchronization issues may indicate more reasonably extraneous load (Skulmowski and Xu, 2022; Greer et al., 2022). It is significant, no matter how difficult it might be to do so.

The process data may be utilized to assess if the load-reducing measures are implemented as planned. Segmented practice has also been found in skills-training literature to lead under certain conditions to less error-prone behavior and more efficient behavior compared to whole-task practice (Greer et al., 2022). Similar investigations in Guzheng could compare the effects of segmented rehearsal on early processing load versus merely delaying reintegration. Signs of learners doing more analytical actions, like the isolation of repeated patterns or deliberate rehearsal of structural units, could also be seen as a sign of cautious inference of germane processing and schema-building work (Choi and Lee, 2022; Choi and Kim, 2021). The word that matters here is cautious.

#### 5.2.4 Practical and analytical challenges

Although they have potential, process-data systems are not simple to implement effectively. Integration is an issue. Smart instruments, learning platforms, eye trackers, performance records, and learner journals could be used as important sources of evidence in the research of Guzheng learning, but such sources generate different types of data and work at different time scales (Choi and Kim, 2021; Montgomery et al., 2019). It is challenging both technically and theoretically to integrate these flows into a consistent analytic system.

The issue of ethics and privacy should be considered as well. Practice records in detail, especially when combined with biometric or performance data, can create concern amongst learners and decrease their enthusiasm to take part in full (Choi and Kim, 2021). Clear consent processes, open handling of data, and prudent limits on the gathering of information are not a choice. These are methods.

Another problem relates to interpretation. Learning analytics can generate very predictive models, but predictive power by itself is insufficient when the predictors behind the prediction are incomprehensible (Li et al., 2023). Interpretability is important in instructional research. To be effective, Guzheng teachers should not only be aware that a student will have difficulties, but they should also understand the reasons behind those difficulties and find out the type of assistance that would be useful.

### 5.3 Retention, transfer, and the evaluation of learning outcomes

The significance of measuring cognitive load will eventually be based on its ability to contribute to explaining important learning results. Retention and transfer are the two most important outcomes in the Guzheng education. Retention is concerned with whether learners can reproduce a skill after a period. Transfer is concerned with whether they can use what they learned in other technical or musical settings. Combined, these outcomes serve as a better measure of instruction effectiveness than immediate performance.

#### 5.3.1 Cognitive load and retention

According to CLT, under these conditions of decreased extraneous load and aided germane processing, learners are more prone to create stable schemas that have been present throughout the period (Skulmowski and Xu, 2022). Studies on digital learning environments have documented that better coordinated materials have a higher chance of supporting retention compared to split-attention formats, whereby decreased extraneous load plays a significant intermediary role (Skulmowski and Xu, 2022). This logic applies to Guzheng learning as well, whereby integrated demonstrations and more aligned notation could promote more lasting retention than fragmented materials. However, empirical evidence in Guzheng has not been abundant yet.

The subjective scale might also be useful in this case if germane load is carefully measured. In the simulated learning process, there have been instances where higher germane loads were linked to enhanced retention at a later time, probably due to the fact that learners took some efforts in comprehending the task instead of simply completing it (Greer et al., 2022). Using Guzheng terminology, a student who consciously tries to grasp how a vibrato or plucking pattern can be applied musically might be more likely to remember such a technique than someone who simply emulates it. This is possible. It requires direct testing.

Distribution of practice is important as well. According to the studies on skill acquisition and learning, spaced practice has been shown to be more effective in supporting retention than massed rehearsal in part because it does not cause the overloading of the mind and instead encourages repetition of schemas (DeKeyser, 2020). This means that in the teaching of guzheng, these short and repetitive practice sessions are particularly important areas of focus in future empirical work, and not only a common pedagogical wisdom.

#### 5.3.2 Cognitive load and transfer

The transfer requirement is more challenging to meet than the retention requirement, as it needs learners to use the existing schema flexibly in unfamiliar conditions. In CLT theory, transfer is highly dependent on whether learners have constructed generalized and meaningful representations versus isolated habits (Choi and Lee, 2022). It is why germane load is of great significance. Since learners can take time to comprehend principles, such as how the pressure affects the quality of tone or how rhythmic grouping determines phrasing, they might find themselves in a better position to implement them in new circumstances.

The extraneous load is counteractive to that process as it uses up resources that could have been used in abstraction and schema refinement. The study of multimedia learning has demonstrated that inadequately produced materials are likely to hinder the transfer, even if students do well in the short term (Skulmowski and Xu, 2022). This means that in the case of the Guzheng, students with fragmented notation or redundant multimedia can copy a passage well in the immediate practice but fail to generalize the technique to new repertoire.

Objective indices can also be considered in this case. The physiological measurements like heart rate variability and other similar measurements have been applied to measure cognitive load of complex tasks, and it has been found that less overload during learning can be linked to good performance on later transfer (Minkley et al., 2021; Ayres et al., 2021). Ideally, Guzheng investigators might make use of comparable measures and transfer tasks that require learners to use a familiar approach in another piece, manner, or tone setting. It is not clear whether such measures would be sufficiently specific to music learning.

#### 5.3.3 Schema construction and automation as mediating processes

The correlation between cognitive load and learning results is not straightforward in a simplistic mechanical way. It is influenced by the construction of schema and its automation, both of which can be applied to explain how the work put into learning turns into the subsequent performance (DeKeyser, 2020). The construction of a schema consists of creating structured mental representations that link technique, perception, and musical purpose. Automation decreases the level of conscious control after such representations have been stabilized through practice.

Indicators of germane load can be helpful in part as they can provide indirect information about the energy used to build schemas. Immersive learning research has indicated that individuals who dedicate greater effort to comprehension usually create more coherent representations of the task (Huang et al., 2019). The same trend could be observed in the Guzheng teaching context, where the students attempt to comprehend the relationship between technical action and expressive outcome instead of focusing on individual movements alone. Conjecture, indeed. Fact, not at all.

Contrariwise, automation is made apparent as performance requirements are reached, which previously were demanding of effort but then start to demand less conscious control. The presence of practice logs, error reduction, speed improvement, and decreased perceived effort in a sequence of repeated sessions can indicate that a technique is being automatized (DeKeyser, 2020). This is significant in the process of learning Guzheng since automation has the potential to release cognitive capacities to more advanced musical issues, such as interpretation, time flexibility, and expressive delicacy.

#### 5.3.4 Challenges in evaluating musical outcomes

The retention and transfer of Guzheng learning are harder to measure than in many fields of study, as musical performance is, *per se*, multidimensional. Precision is important; however, time, pitch, synchronization, and emotion are also relevant. It is possible that a technically accurate performance can be musically poor, and vice versa, that a musically strong performance may have slight technical inconsistency. The assessment of outcome is thus more complicated than simply tallying up right answers and wrong answers.

One of the practical solutions is rubrics. Studies of self-assessment and performance evaluation indicate that with appropriate design, rubrics can enhance judgment consistency and decrease ambiguity of assessment (Krebs et al., 2022). The application of rubrics in the study of Guzheng could be useful to generalize the evaluation of the dimensions, including the accuracy of fingers, rhythmic stability, tone quality, and expressive control. It will be simpler to connect the performance results with the measurements of cognitive load without minimizing musical education to a single grade.

There is also a challenge in longitudinal design. The meaningful investigation of retention and transfer requires that learners be evaluated after enough time has elapsed, but subsequent evaluations contribute to attrition rates and create complications with study designs (Choi and Kim, 2021). Digital tools could be useful as they allow remote follow-up, automated reminders, or repeated low-burden assessment, but cannot solve the issue. They just make it less challenging.

#### 5.3.5 Instructional implications

The link between cognitive load, retention, and transfer is pedagogically relevant to Guzheng instruction. When certain types of instructional design decrease unnecessary extraneous load and facilitate schema construction, their usefulness is not restricted to their effectiveness in a single lesson but can also affect the ability of learners to retain and generalize information over time. This provides some careful backing to a few practical implications that have been suggested by the overall literature: improved incorporation of notation and fingering signals, increased usage of spaced practice, enhanced focus on analytical interaction with patterns and techniques, and feedback that is rubric-based that encourages self-monitoring (Skulmowski and Xu, 2022; Choi and Lee, 2022; DeKeyser, 2020; Krebs et al., 2022).

To sum up, the highest level of measuring cognitive load in learning Guzheng is achieved when it is measured using a combination of methods. Each of the subjective scales, process data, physiological indicators, and performance outcomes represents some part of the picture. Combined, they can help to not just understand if an instructional strategy seems effective, but also if it alters the circumstances in which learning is made more sustainable, efficient, and transferable.

## 6 Design implications for innovative Guzheng learning environments

The previous paragraphs imply that the cognitive load involved in Guzheng learning is influenced by the demands of the instrument as well as how information, demonstration, feedback, and practice are organized around the learner. It can be expressed easily, but it is significant. Learning difficulties in Guzheng instruction cannot be attributed solely to repertoire or technique, but to the design of the learning environment itself. Thus, the enhancement of the guzheng learning environment is more focused on minimizing unnecessary fragmentation instead of increasing the number of tools and aiding the learners to focus their attention on musically meaningful processing.

### 6.1 Notation and multimodal presentation design

The notation is an essential interface of instruction and performance of the Guzheng studies. It is also one of the areas where avoidable extraneous load may grow rapidly. According to the CLT point of view, in such cases, students do not need to integrate the information alone, but it should be given to them in a more unified manner. If fingerings, cues, rhythmic structure, expressive markings, and melodic content are scattered in disconnected places, a portion of the learner’s limited cognitive processing is used to coordinate the display instead of understanding or playing the music (Sweller et al., 2019; Skulmowski and Xu, 2022).

An implication that can be taken practically is simple; it is important to minimize any unnecessary redundancy where applicable. In case finger cues may be written in the notation directly, instead of being spread over individual columns or remote annotations, split attention is probably going to go down (Sweller et al., 2019; Skulmowski and Xu, 2022). This is especially true with novice learners who have not necessarily developed steady schemas that tie notation with movement. Similarly, highly interactive passages may also be presented better if they are divided into smaller and more feasible pieces. The passage is not simple just because of that. It makes initial processing less crowded, though (Paas and Merriënboer, 2020).

The concept of visual hierarchy also plays an important role. Dense notation can be more useful to learners when the most important information is readily foregrounded. Contrast, restrained color use, or selective highlighting could serve to focus attention on the musically or technically dominant part so long as these signals are used in a consistent manner without becoming a new source of distraction (Zhang Y. et al., 2024; Zhang S. et al., 2024). Visual and emotional design features may also shape learners’ affective and cognitive responses, although they should be used cautiously to avoid creating new distractions (Liew et al., 2022). The same caution should be taken with multimodal support. Fingerboard diagrams, string labels, or movement prompts might support understanding when they are closely aligned with the corresponding phrase (Korbach et al., 2019; Ginns and Kydd, 2019); however, when too many cues are used, they may create another source of visual competition (Bae, 2024).

The level of expertise of the learner should also be considered. What is helpful to a beginner might be useless or even annoying to a more advanced learner, particularly when explicit cues just repeat what is accessible to them in long-term memory (Sweller et al., 2019). The notation must not, however, be seen as a rigid form. A beginner may require explicit fingering, segmentation, and tempo instructions. A more advanced learner may need space. Excessive visual assistance may also become a kind of burden.

### 6.2 Video-mediated demonstration and sequencing

Video can preserve movement, timing, and sound in ways that static notation cannot. Its educational value, however, depends on the coordination of these elements. Hand movement, sound production, and camera framing should be synchronized so that learners can process them as parts of the same performance event (Sweller et al., 2019; Skulmowski and Xu, 2022). Close-up views may be useful for plucking technique, left-hand pitch shaping, or transitions between hand functions, whereas rapid camera changes and dense overlays may compete for attention.

Long demonstrations can provide an overview of complete performance, but shorter, goal-focused clips are generally more suitable for initial practice (Costley et al., 2020). A clip may isolate one technique, phrase, or coordination problem and allow learners to pause or replay the relevant material. This use of segmentation should remain linked to the complete passage so that learners can later reconnect the observed component with whole-task performance. Long demonstrations can provide an overview of complete performance, but shorter, goal-focused clips are generally more suitable for initial practice (Costley et al., 2020; Rekik et al., 2021; Yu, 2021).

Video support may also be faded as familiarity increases. Early clips can include slowed modeling, brief explanations, or selective visual markers; later clips can reduce these prompts and allow more independent observation (Janson et al., 2019; Ou et al., 2025). Interactive features should be used selectively and positioned outside demanding performance sequences where possible (Skulmowski and Xu, 2022; Sidi and Ackerman, 2024). The aim is to direct attention to relevant movement and sound rather than to increase the number of information layers.

### 6.3 Classroom workflow and guided practice design

CLT is not limited to materials but has been extended to the organization of classroom practice. Classroom workflow in Guzheng education may be used to manage difficulty in a productive manner or increase it in an unnecessary way. A cognitively informed workflow is not merely a way of sequencing activities; it organizes goals, demonstrations, practice cycles, feedback, and reflection so as to make challenging material easier to handle with time.

Prior to practice, the instructional objectives have to be clear and concise so that they can still be workable. Students are more likely to distribute their efforts better if they understand whether the focus of the session is on right-hand plucking pattern, left-hand expressive technique, or both of them in a short phrase (Sweller et al., 2019). The worked-example demonstration can be especially helpful during this step since it offers an example of the performance prior to asking learners to perform it. With the combination of demonstration and segmentation with temporary scaffolding, there might be fewer chances of students feeling overloaded at the beginning of practice (Janson et al., 2019; Ou et al., 2025).

While practicing, conscious attention is a better strategy than repetition by itself. There is value in repetition, but some kinds of repetition are not as valuable as others. Clear technical or expressive problems, specific feedback, and modification of effort to attain manageable performance goals are the most beneficial aspects of learners working on these types of problems (Ou et al., 2025). This monitoring of cognitive load may also be used to assist in this process, even when high-tech monitoring devices are unavailable. The eye-tracking technique and the EEG can provide useful information in the context of research, but in classrooms, teachers do not generally use such complex indicators: repeated breakdown, obvious frustration, loss of fluency, or self-reporting of overload (Hawthorne et al., 2019; Ayres et al., 2021). These signs are important. These signs frequently arise prior to the establishment of failure.

The concept of group work may also prove valuable in certain situations. Peer feedback, paired practice, or ensemble-based exercises may share some of the information load and provide other reflections, so long as the collaboration does not detract but enhances the targeted skill (Janssen and Kirschner, 2020). Such a qualification is worth noting. Collaboration in instrumental learning does not necessarily have positive effects; at times it clarifies, and at other times it fractures. It greatly depends on time, the type of tasks, and the preparedness of learners.

The practice, reflection, and transfer are also significant after practice. It is important that learners have the chance to assess what has improved, what is still unstable, and how a skill can be applied to a new musical setting. The progress made may become clearer through practice logs, self-assessment, and aided reviewing of recordings over time (Wirth et al., 2020). Transfer tasks are particularly beneficial since they facilitate encouraging learners to go beyond isolated repetition and use emergent schemas in new compositions, tempo, or expressive contexts (Sweller et al., 2019). A good workflow is not concluded by repetition; it should be completed by reapplication.

### 6.4 Digital resource configuration and adaptive support

Digital resources are most useful when they coordinate notation, demonstration, practice, and feedback around a clearly defined learning task. Platforms containing many disconnected functions may create navigation demands that compete with musical practice. By contrast, linking a musical phrase directly to its corresponding demonstration or feedback may reduce fragmentation and support more coherent practice (Skulmowski and Xu, 2022).

AI-supported systems may personalize task difficulty, feedback timing, or the amount of scaffolding by analyzing recurring errors or practice patterns (Choi and Kim, 2021; Ou et al., 2025). For example, a system could temporarily reduce tempo, isolate a difficult passage, or provide an additional demonstration. Such responsiveness is potentially useful, but direct evidence from Guzheng learning remains limited, and inaccurate or intrusive feedback may itself increase cognitive load.

VR and AR may provide simulated performance settings or positional guidance within the learner’s field of action (Buchner et al., 2021). Their educational value depends on whether they simplify perception–action coordination without adding unnecessary interface or sensory demands. Digital tools should therefore be evaluated according to their effects on attention, cognitive load, and learning outcomes rather than according to their technical complexity. Table 4 summarizes these evidence-informed design considerations.

**Table 4:** Design factors that might be considered when creating innovative Guzheng learning environments in relation to cognitive load.

Design domain	Main issue	Possible design consideration	Potential pedagogical implication	References
Notation design	Split attention, visual clutter, and weak score–technique linkage	Place fingerings and technical marks as near as possible to their corresponding visual area; apply segmentation and a moderate visual hierarchy when applicable	It can decrease the unnecessary extraneous load and help in providing a better correspondence between notation and behavior, particularly for novice learners	Sweller et al. (2019); Skulmowski and Xu (2022); Paas and Merriënboer (2020); Zhang et al. (2024); Bae (2024)
Video-mediated demonstration	Overlong demonstrations, weak synchronization, and dense overlays	Shorter, goal-oriented clips are more effective; ensure that sound and movement go together well; eliminate explanatory support slowly with the growth in learner familiarity	May improve perceptual access to movement and reduce unnecessary processing pressure during early learning	Sweller et al. (2019); Skulmowski and Xu (2022); Janson et al. (2019); Costley et al. (2020); Ou et al. (2025); Sidi and Ackerman (2024)
Classroom workflow	Unclear goals, poorly sequenced tasks, and overload during practice	Use clear but achievable objectives, demonstrate prior to implementation, directed segmentation, and chances of reflection after training	May support a more manageable progression from observation to practice and later reapplication	Sweller et al. (2019); Hawthorne et al. (2019); Janson et al. (2019); Ou et al. (2025); Ayres et al. (2021); Janssen and Kirschner (2020); Wirth et al. (2020)
Adaptive digital support	Learners differ in expertise, pace, and overload threshold	Adjust difficulty, feedback timing, and scaffolding intensity in response to learner performance where feasible	May improve alignment between task demands and learner readiness under some conditions	Choi and Kim (2021); Ou et al. (2025); Ayres et al. (2021)
Multimedia platforms	Fragmented resources and navigation burden	Coordinate notation, demonstration, and feedback more closely within the same practice environment, where possible	May reduce fragmentation and support more coherent self-paced practice	Skulmowski and Xu (2022); Choi and Kim (2021); Song (2025)
AR/VR-enhanced guidance	Possible novelty load, realism effects, and interface burden	Apply AR/VR in a selective manner as a support of position, a context of rehearsal, or an embodiment of guidance; do not use interfaces that are unnecessarily complex	Can be used to support embodied rehearsal under certain conditions, as long as additional sensory or interface requirements are kept low	Buchner et al. (2021); Oje et al. (2025); Skulmowski and Rey (2020)

This table summarizes design considerations discussed in the review from a cognitive load perspective. The implications should be interpreted cautiously. Direct Guzheng-specific evidence remains limited for several entries, and some considerations are informed by broader music-learning or adjacent complex-skill literature. The table should therefore be read as a set of evidence-informed considerations rather than as validated instructional prescriptions.

## 7 Research gaps and agenda for future studies

Even though the literature covered in this review offers a great foundation to talk about cognitive load during the process of learning Guzheng, the evidence base is unbalanced. That inequality ought to be made clear. Most of the existing discourse can be theoretically consistent and pedagogically meaningful, but practical empirical research in Guzheng teaching is sparse. In the current situation, the discipline depends on the layered evidentiary framework: a small amount of Guzheng-related work, a larger amount of music and instrumental-learning studies, and a third layer of results based on the related complex-skill areas. These sources have been handled with care in this review, but there is work to be done that will close this gap. The biggest problem is not multiplying concepts. Instead, it should be to examine, in the context of Guzheng education, whether the instructional mechanisms analyzed in this review are likely to behave as they should in an actual classroom setting.

### 7.1 Domain-specific gaps in Guzheng research

The issue of domain-specificity is a longstanding gap. The majority of the conceptual background of the current review was influenced by general CLT research, multimedia learning, more extensive research on music learning, simulation-based training, and related fields of complex-skill learning. Although these sources are useful, they can also not be used as the basis of evidence of the Guzheng. An illustration of this is that it is still uncertain how much the results of segmentation, notation integration, or feedback timing are applicable to contexts in which left-hand expressive control, right-hand sequencing, and culturally specific notation practices operate in very special ways. That is exactly why the discipline is still weak.

The problem is well explained by the notation design. Available literature implies that segmentation and integration may be used to minimize avoidable load in complex learning environments (Paas and Merriënboer, 2020), but it has not been conclusively shown how they may impact Guzheng notation. Research evaluating the effectiveness of an integrated notation as opposed to a non-integrated notation, or a segmented notation as compared to a non-segmented notation of technical dense excerpts would thus be particularly useful. This also works in the case of demonstration. One could speculate that extraneous load in the initial stages of learning can be decreased using carefully synchronized video, slowed modeling or selective cueing, but there is little Guzheng-based evidence to support this. The practice that seems pedagogically sound at the moment requires domain-specific verification.

### 7.2 Feedback, technology, and learner variation

A second research gap concerns whether feedback and technology-supported instruction operate differently across learners and tasks. Immediate feedback may assist early motor correction, whereas delayed feedback may allow more self-evaluation and reflection (Sweller et al., 2019; Ou et al., 2025). However, the appropriate timing, modality, and intensity may depend on expertise, prior instrumental experience, working-memory capacity, and familiarity with digital tools.

The same uncertainty applies to adaptive systems and immersive technologies. Current evidence does not establish whether AI-, VR-, or AR-supported instruction reduces cognitive load more effectively than conventional teaching in Guzheng contexts (Choi and Kim, 2021; Ou et al., 2025; Buchner et al., 2021). Future studies should compare these forms of support using common outcome measures and should examine whether their effects are moderated by learner characteristics, task complexity, and interface design. Such work is needed before technology-specific recommendations can be made.

### 7.3 Retention, transfer, and longitudinal evidence

The third gap deals with the concept of time. Most of the existing literature, both within music education and outside it, continues to rely largely on short-term results. However, Guzheng learning is cumulative, and no judgment of instructional effectiveness can be made without questioning whether or not learners are able to retain the techniques over time, as well as transfer them between compositions, tempos, and expressive contexts. Fluency at once is informative, but it does not suffice. The performance in the moment of a learner can be high, and yet the learner does not develop a steady and versatile schema.

Longitudinal designs are thus necessary. The most convincing evidence on whether load-reduction strategies can be used to build long-lasting learning will be provided by studies that involve delayed retention tests, follow-up performance assessments, or transfer tasks (Sweller et al., 2019). It is particularly crucial in the case of the mechanisms highlighted in the current review: segmented practice, worked examples, and feedback scaffolding, because they have the potential of enhancing short-term manageability without necessarily leading to later independence. Future studies should consequently not only measure performance accuracy, but also the slow evolution of automation, expressive control, reintegration, and adaptability over time.

### 7.4 Methodological priorities for future empirical work

In terms of methodology, future Guzheng studies should incorporate a better combination of various data sources. The mixed-methods designs are particularly attractive since it enables researchers to integrate the outcomes of the performance with the experience of the learner and interpretation of the context (Skulmowski and Xu, 2022). Subjective scales are still relevant, but they ought to be supplemented by objective measures like physiological parameters, eye-tracking, traces of automatic performance, or any other form of behavioral data whenever possible (Ayres et al., 2021). This is not to give any special value to instrumentation itself. Rather, this is to provide more defensible inferences about cognitive load.

The adaptive assessment tools should also receive consideration. It might be feasible to measure hand-position accuracy, tempo stability, and consistency of movement with the help of AI-assisted video analysis, computer vision, or sensor-based tracking more objectively than using conventional observation alone (Ou et al., 2025). But those tools are to be read cautiously and related to significant musical results instead of being considered as enough on their own. The fact that something is measured accurately does not necessarily mean that it will be pedagogically relevant.

Cross-cultural validation is also a priority. The teaching of Guzheng music has its origins in the Chinese musical and educational heritage, and many of the more comprehensive theoretical perspectives that are employed in this review were conceived within the Western research environment. Future studies ought thus consider checking whether cognitive load regulation strategies are applicable in the same way to all learner populations, cultural environments, and pedagogical practices, or whether they assume a uniform applicability (MacRitchie et al., 2020). This problem is not marginal. It directly touches upon how much the existing theory can go without being reinterpreted.

### 7.5 A focused agenda for future research

The current review implies that there are five immediate priorities. Firstly, segmentation and integration as variables relating to notation design need to be evaluated experimentally in Guzheng learning conditions instead of being derived by extrapolation out of related fields. Secondly, comparisons between immediate and delayed feedback need to be made at various levels of learner experience and between various levels of task difficulty. Thirdly, the impact of AI-supported systems on cognitive load, skill performance, and learner autonomy compared with more traditional types of support ought to be explored in future studies. Fourth, they ought to determine how learner attributes such as expertise, previous instrument experience, and cognitive ability influence the effects of instructional design. Fifth, they must evaluate the role of VR and AR tools in improving the performance not only at once but also in the future, reintegration, and performance confidence.

**Figure 2:**
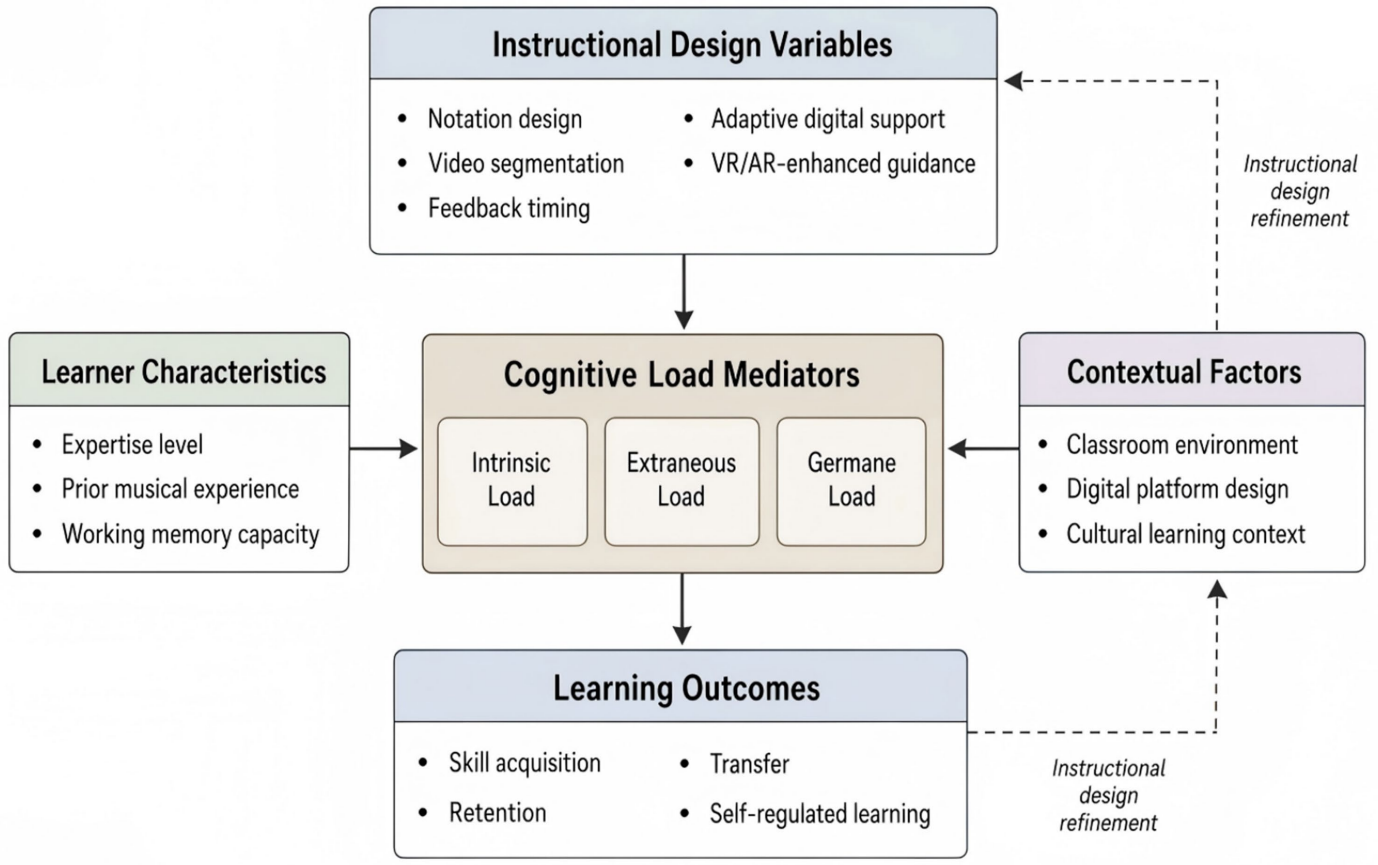
The research agenda of future empirical studies about cognitive load optimization in the process of learning Guzheng.

These priorities are not a fixed framework and do not imply that every potential idea would be able to withstand direct testing. This uncertainty is precisely why direct empirical testing is required. They are valuable because they shift the debate beyond the general theoretical possibility to something more defendable in terms of design knowledge about Guzheng education. In Figure 2, the research agenda suggested as the basis of future empirical research is presented, and the relationships between instructional design variables, cognitive load mediators, moderating factors, and learning outcomes in Guzheng learning are specified.

The diagram presents the suggested research program based on the literature review. The model sees instructional design variables as the main causes of learning conditions, working via three cognitive load mediators, intrinsic, extraneous, and germane loads, to determine the effect of learning conditions on learning outcomes, including skill acquisition, retention, transfer, and self-regulated learning. It also recognizes learner characteristics and contextual factors as significant moderators that can affect how instructional interventions operate in various contexts of Guzheng learning. The framework shows that future empirical research is needed that will not only test the effectiveness of particular instructional strategies but also who they are effective with, when, and by what cognitive processes they enhance learning.

## 8 Conclusion

This narrative review used Cognitive Load Theory to examine Guzheng learning as a form of complex skill acquisition. Learners must coordinate notation, timing, bimanual movement, auditory monitoring, and musical interpretation within a limited working-memory capacity. The difficulty of this process reflects both the intrinsic complexity of performance and the way instruction organizes information and practice.

The reviewed literature suggests that split attention, poorly coordinated demonstrations, and redundant multimedia input may add avoidable extraneous load (Sweller et al., 2019; Skulmowski and Xu, 2022). Demonstration-guided practice, segmented rehearsal followed by reintegration, and feedback adjusted to the learner and task appear pedagogically relevant (Mirza et al., 2019; Luca et al., 2021; Fyfield et al., 2019). These conclusions remain provisional because much of the supporting evidence comes from broader music learning and adjacent complex-skill fields rather than direct Guzheng studies.

The review also indicates that instructional support should be evaluated by its cognitive function rather than by its format. Notation, video, adaptive systems, and immersive technologies may be useful when they direct attention and coordinate relevant information. They may be less useful when they introduce additional navigation, interface, or sensory demands. Their effects in Guzheng education require direct empirical testing.

Overall, Guzheng learning provides a culturally situated case for examining how cognitive load, instructional sequencing, scaffolding, and feedback shape complex artistic skill acquisition. Future studies should test these mechanisms in direct Guzheng settings and should assess retention, transfer, learner variation, and independent performance over time. The present review offers a cautious foundation for that work rather than a fixed set of instructional prescriptions.
